# Hormone-Responsive BMP Signaling Expands Myoepithelial Cell Lineages and Prevents Alveolar Precocity in Mammary Gland

**DOI:** 10.3389/fcell.2021.691050

**Published:** 2021-07-15

**Authors:** Chunlei Shao, Pengbo Lou, Ruiqi Liu, Xueyun Bi, Guilin Li, Xu Yang, Xiaole Sheng, Jiuzhi Xu, Cong Lv, Zhengquan Yu

**Affiliations:** State Key Laboratories for Agrobiotechnology and Key Laboratory of Precision Nutrition and Food Quality, Ministry of Education, Department of Nutrition and Health, College of Biological Sciences, China Agricultural University, Beijing, China

**Keywords:** mammary gland development, BMPR1a, p63, Slug, alveolar precocity

## Abstract

Myoepithelial and luminal cells synergistically expand in the mammary gland during pregnancy, and this process is precisely governed by hormone-related signaling pathways. The bone morphogenetic protein (BMP) signaling pathway is now known to play crucial roles in all organ systems. However, the functions of BMP signaling in the mammary gland remain unclear. Here, we found that BMPR1a is upregulated by hormone-induced Sp1 at pregnancy. Using a doxycycline (Dox)-inducible BMPR1a conditional knockout mouse model, we demonstrated that loss of BMPR1a in myoepithelium results in compromised myoepithelial integrity, reduced mammary stem cells and precocious alveolar differentiation during pregnancy. Mechanistically, BMPR1a regulates the expression of p63 and Slug, two key regulators of myoepithelial maintenance, through pSmad1/5-Smad4 complexes, and consequently activate P-cadherin during pregnancy. Furthermore, we observed that loss of BMPR1a in myoepithelium results in the upregulation of a secreted protein Spp1 that could account for the precocious alveolar differentiation in luminal layer, suggesting a defective basal-to-luminal paracrine signaling mechanism. Collectively, these findings identify a novel role of BMP signaling in maintaining the identity of myoepithelial cells and suppressing precocious alveolar formation.

## Introduction

The mammary gland is a unique organ that develops mostly postnatally (Richert et al., 2000). Its development undergoes complicated epithelial remodeling from puberty to pregnancy, lactation, and involution (Macias and Hinck, 2012). The mammary epithelium includes an outer layer of basal/myoepithelial cells, expressing K5/K14, and an inner layer of luminal cells, expressing K8/K18, which maintain a state of dynamic balance (Inman et al., 2015). During pregnancy, both myoepithelial and luminal cells rapidly expand and undergo functional changes that are regulated by hormones; luminal cells differentiate into milk-producing cells to allow lactation, whereas myoepithelial cells expand and contract to squeeze milk throughout the ductal tree (Hennighausen and Robinson, 2005). However, the mechanism by which myoepithelial cells expand during pregnancy in response to hormones is not fully understood.

The myoepithelial cells of the mammary gland include mammary stem cells, which are capable of regenerating an epithelial tree composed of myoepithelial and luminal lineages (Shackleton et al., 2006; Stingl et al., 2006). Previous studies have shown that p63 and Slug maintain the stemness of mammary stem cells and govern the fate of mammary myoepithelial cells. A p63 isoform, ΔNp63, promotes mammary stem cell activity by interacting with WNT signaling (Chakrabarti et al., 2014; Lindley et al., 2015; Sreekumar et al., 2017). p63 is required for maintaining mammary epithelial cells in a myoepithelial state (Ding et al., 2019). Sustained expression of p63 promotes embryonic multipotent progenitors into unipotent mammary basal epithelial cells and it could also reprogram adult luminal cells into basal epithelial cells (Wuidart et al., 2018). Yalcin-Ozuysal et al. (2010) found that ΔNp63 and Notch antagonistically interact to maintain myoepithelial identity. Slug is another important regulator of cell state transition, which maintains mesenchymal features of myoepithelial cells (Lamouille et al., 2014). Loss of Slug results in decreased mammary stem cell activity and compromised DNA damage repair in myoepithelial cells (Guo et al., 2012; Gross et al., 2019). Slug interacts with LCD1 to maintain mammary epithelial cells in a myoepithelial-like state and inhibit luminal differentiation (Phillips et al., 2014). Thus, p63 and Slug are essential for the maintenance of myoepithelial integrity.

Bone morphogenetic protein (BMP) signaling plays critical roles in development and disease (Zhang and Li, 2005; Rodriguez et al., 2010). BMP2/4, which are major BMPs, can bind to BMP receptors that transduce signaling through Smad-dependent pathway (Heldin et al., 1997; Feng and Derynck, 2005). When BMP signaling is activated, Smad1/5/8 are phosphorylated and form a complex with Smad4, which then enters to the cell nucleus and activates the transcription of target genes (Massagué, 1998; Qi et al., 2017). It has been implicated that BMP receptors including BMPR1a, BMPR1b and BMPR2 play important roles in the mammary gland development and breast cancer. The activity of BMPR1B regulates the luminal differentiation of mammary progenitors and contributes to the formation of luminal tumor (Chapellier et al., 2015). Disruption of BMPR2 signaling could accelerate of tumor progression with increase of lung metastases in MMTV-PyVT mouse model (Owens et al., 2012). Of these receptors, BMPR1a mediates many basic biological processes, including cell proliferation, migration, differentiation and cell-cell interaction (Ying et al., 2003; Langenfeld et al., 2006; Huang et al., 2014). Global deletion of *Bmpr1a* results in embryonic lethality (Mishina et al., 1995). Studies have reported that BMPR1a regulates neural stem cell fate, skeletal development, and differentiation of hair lineage (Genander et al., 2014; Lee et al., 2018; Wegleiter et al., 2019). During embryonic mammary development, PTHrP upregulates BMPR1a expression to stimulate the outgrowth of mammary buds (Hens et al., 2007; Robinson, 2007). However, due to embryonic lethality, the roles of BMPR1a and BMP signaling in mammary gland development during pregnancy have not been explored.

Here, we found that BMPR1a is upregulated by Sp1 in response to hormones during pregnancy. Using a spatiotemporal myoepithelium-specific BMPR1a-knockout mouse model, we demonstrate that BMPR1a plays important roles in expanding myoepithelial cells and suppressing precocious alveolar differentiation by regulating p63 and Slug at pregnancy.

## Materials and Methods

### Mice

*Bmpr1a*^*flox*^ mice were obtained from Dr. Sarah Millar laboratory at University of Pennsylvania with permission of Dr. Yuji Mishina. *K14-rtTA* (JAX stock no: 007678), *teto-Cre* (JAX stock no: 006234) and *Rosa26-mTmG* (JAX stock no: 007676) transgenic mice were purchased from the Jackson Laboratory. The genetic background of the above transgenic mice is C57BL/6, and littermates were used as controls in this study. NOD-SCID mice and wild-type C57BL/6 mice were purchased from Beijing Vital River Laboratory Animal Technology Company. Genomic DNA was isolated from tail tissue, and PCR was performed using the following primers. Only female mice were used for analysis in this study. For *Cre* induction, 2 mg/mL doxycycline (Dox, D9891, Sigma, St. Louis, MO, United States) and 10 mg/mL sucrose were added to the drinking water, and Dox treatment was performed until sample collection. All mice were fed under specific pathogen-free (SPF) conditions with 12 h of alternating light and dark, temperature was 23 ± 2°C. Primers (5′-3′) for genotyping are listed in Supplementary Table 1.

### Cell Culture and Treatment

HC11 mouse mammary epithelial cells were purchased from Procell Life Science & Technology Co., Ltd (Wuhan, China). HC11 cells were cultured at 37°C with 5% CO_2_ in RPMI-1640 (Gibco, Grand Island, NY, United States) medium supplemented with 10% FBS (Gibco), 1% penicillin/streptomycin (Gibco), 5 μg/mL insulin (Sigma) and 10 ng/mL epithelial growth factor (EGF, Gibco). BMP4 (Abcam, Cambridge, MA, United States) is obtained from *Escherichia coli*, reconstituted in 10 mM citric acid and suitable for functional studies. BMP2 (PeproTech, Rocky Hill, NJ, United States) is obtained from *Escherichia coli*, reconstituted in deionized water containing 0.1% BSA and biological activity verification. For BMPs treatment, HC11 cells were serum starved overnight and then treated with BMP4 (50 ng/mL) or BMP2 (50 ng/mL) at the indicated time points. 10 mM citric acid was used as vehicle control for BMP4, deionized water containing 0.1% BSA was used as vehicle control for BMP2. For hormone stimulation, HC11 cells were grown to 70% confluence and serum starved overnight, then stimulated with 100% ethanol-dissolved 10 nM β-estradiol (E2, Sigma) and/or 100 nM progesterone (Pg, Sigma), 100% ethanol was used as vehicle control, the cells were harvested at 24 h after this treatment.

### Cell Transfection

HC11 cells were grown to 70% confluence and washed twice with Dulbecco’s Phosphate Buffered Saline (DPBS, Gibco), then changed into Opti-MEM (Gibco) medium for 2 h. siRNA or plasmids were transfected using Lipofectamine stem reagent (Invitrogen, CA, United States) according to the manufacturer’s instructions. After 24–48 h of transfection, the cells were collected for further experiments. The pcDNA 3.1 empty vector and pcDNA 3.1-Sp1 overexpression vector were purchased from GenePharma company (Suzhou, China). The siRNA sequences and sh*Bmpr1a* target sequences are listed in Supplementary Table 2.

### Flow Cytometry and Sorting Assays

Mammary glands were dissected from 10- to 12-week-old female mice at pregnancy day 14.5, cut into pieces, and placed in DMEM/F12 (Gibco) with 5% FBS (Gibco), 300 U/mL collagenase (Gibco) and 100 U/mL hyaluronidase (Sigma) for 2 h at 37°C. After digestion, red blood cells were removed with NH_4_Cl (STEMCELL Technologies, Vancouver, BC, Canada), and then a single-cell suspension was obtained by sequential dissociation in 37°C preheated 0.25% trypsin (Gibco) for 2 min and in 5 mg/mL Dispase (STEMCELL Technologies) containing 0.1 mg/mL DNase I (Sigma) for 5 min with pipetting, followed by filtration through a 40 μm filter (BD, San Jose, CA, United States). The single-cell suspension was stained with specific antibodies in DPBS containing 2% FBS for 15 min on ice. After being washed three times with DPBS containing 2% FBS, cells were analyzed by FACS Verse or sorted by FACS Aria. The following antibodies were used: CD45-APC (17-0451-82, eBioscience, San Diego, CA, United States), CD31-APC (17-0311-82, eBioscience), TER119-APC (17-5921-82, eBioscience), CD24-PE-Cy7 (25-0242-82, eBioscience), CD29-FITC (11-0291-82, eBioscience), CD61-PE (561910, BD), and Fixable Viability Dye eFluor 450 (65-0863-14, eBioscience). Data analysis was performed with FlowJo 7.6.1.

### Ovariectomy and Hormone Stimulation

Ten-week-old WT female C57BL/6 mice were bilaterally ovariectomized and allowed to recover for 14 days. For hormone stimulation, the mice were induced by intraperitoneal injections once every other day with E2 (5 μg per time) and Pg (3.5 mg per time) for consecutive 21 days. The control mice were intraperitoneally injected with corn oil.

### Transplantation Assay

Basal cells (Lin^–^CD24^+^CD29^*high*^) were FACS sorted from 6-week-old female control and *K14-rtTA;teto-Cre;Bmpr1a^*fl/fl*^* mice and resuspended in a 1:1 DPBS/Matrigel (BD) solution at a concentration of 2,000 cells per 10 μL. Cells and Matrigel solutions were injected in 10 μL into 3-week-old female NOD-SCID mice which cleared fat pads of the inguinal mammary glands. After transplantation, all mice received water with 2 mg/mL Dox and 10 mg/mL sucrose immediately. Eight weeks after transplantation, mouse inguinal mammary glands were analyzed. Ductal outgrowths were detected under dissection microscope (Leica, Wetzlar, Germany).

### Histology and Immunostaining

Mammary glands were fixed in 4% PFA, embedded in paraffin and sectioned at 5 μm. For histological analysis, the sections were dewaxed in xylene and rehydrated with serial dilutions of ethanol. The sections were stained with hematoxylin (Sigma) for 10 min and eosin (Sigma) for 10 s. For immunohistochemistry staining, antigen retrieval was performed by heating slides for 20 min in 0.01 M citrate buffer (pH 6.0) in a microwave. The sections were subjected to natural cooling and immunostained by following the SP Kit (ZSGB-Bio, Beijing, China) manufacturer’s instructions. For immunofluorescence staining, the sections were blocked with blocking solution (Beyotime, Shanghai, China) for 1 h at room temperature after antigen retrieval and then incubated with primary antibodies at 4°C overnight. The sections were washed three times in phosphate buffer saline (PBS, pH 7.4), incubated with secondary antibodies for 1 h at room temperature, and then counterstained with 2-(4-amidinophenyl)-6-indolecarbamidine dihydrochloride (DAPI, Beyotime) for 8 min. For immunocytochemistry staining, cells were fixed in 4% PFA for 15 min, washed three times with PBS and blocked with blocking solution for 1 h at room temperature. Then, the cells were incubated with primary antibodies at 4°C overnight, incubated with secondary antibodies for 1 h at room temperature, and counterstained with DAPI for 8 min. TUNEL (Beyotime) staining was performed according to the manufacturer’s instructions. Images were acquired under the Leica microscope photograph system (Leica). The following primary antibodies were used: BMPR1a (1:250, 12702-1-AP, Proteintech, Rosemont, United States), Sp1 (1:500, 21962-1-AP, Proteintech), K14 (1:400, ab7800, Abcam), K8 (1:800, ab59400, Abcam), pSmad1/5 (1:800, 9516, CST, Danvers, MA, United States), WAP (1:400, sc-374648, Santa Cruz, CA, United States), β-Casein (1:400, sc-166530, Santa Cruz), Plin2 (1:500, ab108323, Abcam), Stat5 (1:200, ab68465, Abcam), pStat5 (1:400, 9359, CST), Prlr (1:100, ab170935, Abcam), Ki67 (1:800, ab15580, Abcam), Cyclin D1 (1:1000, 55506, CST), Cleaved caspase3 (1:1000, 9664, CST), p63 (1:1000, ab124762, Abcam), Slug (1:400, 9585, CST), Smad4 (1:800, 46535, CST), P-cadherin (1:200, AF761, R&D, Minneapolis, MN, United States), GFP (1:1000, ab290, Abcam), BMP4 (1:200, MAB5020, R&D) and Integrin β3 (1:250, 13166, CST). The following secondary antibodies were used: Alexa Fluor 488 and 594 goat anti-mouse or anti-rabbit IgG (H + L) and Alexa Fluor 488 donkey anti-goat IgG (H + L) (1:400, Invitrogen).

### Whole-Mount Staining of Mouse Mammary Glands

Fresh inguinal mammary glands were spread on glass slides, fixed in Carnoy’s fixative (ethanol: chloroform: glacial acetic acid, 6:3:1) for 24 h at room temperature, immersed in 70, 35, and 15% ethanol for 15 min each, immersed in deionized water for 5 min, and stained in carmine alum for 20 min. Next, the mammary glands were washed in 70, 95, and 100% ethanol for 15 min each, cleared in xylene for 2 days and mounted.

### RNA Extraction and qRT-PCR Analysis

Adipocytes and red cells were removed from inguinal mammary glands, or myoepithelial cells (Lin^–^CD24^+^CD29^*high*^) and luminal cells (Lin^–^CD24^+^CD29^*low*^) were FACS-sorted from mouse mammary glands. Total RNA was extracted from cells or mouse mammary gland epithelial cells using TRIzol reagent (Thermo Scientific, Waltham, MA, United States) following the manufacturer’s instructions. RNA was converted to complementary DNA using the MMLV cDNA synthesis kit (Promega, WI, United States). qRT-PCR was performed using the SYBR green detection system (Roche, Mannheim, Germany). *Gapdh* served as an internal control. Quantification of gene expression level was based on cycle threshold (Ct) value, relative expression of mRNA was calculated using the 2^–Δ^ ^Δ^ ^*Ct*^ method. qRT-PCR primers (5′-3′) are listed in Supplementary Table 3.

### Western Blotting Analysis

Protein was isolated from mammary gland epithelial cells and cell lines using RIPA lysis buffer (Beyotime) with protease inhibitor cocktail (Beyotime). Protein concentrations were measured using a BCA protein assay kit (Beyotime) following the manufacturer’s instructions, and then the proteins were denatured with loading buffer. A 30 μg total protein was electrophoresed by 8 or 10% sodium dodecyl sulfate-polyacrylamide gel electrophoresis (SDS-PAGE) and transferred to polyvinylidene difluoride (PVDF) membranes (Millipore, Bedford, MA, United States). The PVDF membranes were blocked in 5% non-fat dry milk for 1 h at room temperature and incubated with primary antibodies at 4°C overnight. Then, the membranes were incubated with horseradish peroxidase (HRP)-linked secondary antibodies (1:5000, Beyotime) for 1 h at room temperature. Protein expression was visualized using a chemiluminescent detection system. The following primary antibodies were used: BMPR1a (1:1000, 12702-1-AP, Proteintech), Sp1 (1:2000, 21962-1-AP, Proteintech), pSmad1/5 (1:1000, 9516, CST), β-Casein (1:500, sc-166530, Santa Cruz), Plin2 (1:1000, ab108323, Abcam), Stat5 (1:1000, ab68465, Abcam), pStat5 (1:1000, 9359, CST), K14 (1:1000, ab7800, Abcam), p63 (1:1000, ab124762, Abcam), Slug (1:1000, 9585, CST), K8 (1:1000, ab59400, Abcam), P-cadherin (1:1000, AF761, R&D), GAPDH (1:1000, 5174, CST), β-Actin (1:1000, 4970, CST), and β-Tubulin (1:5000, ab6046, Abcam). Western blotting data were quantified by Image J software.

### Dual Luciferase Activity Assays

**FIGURE 1:**
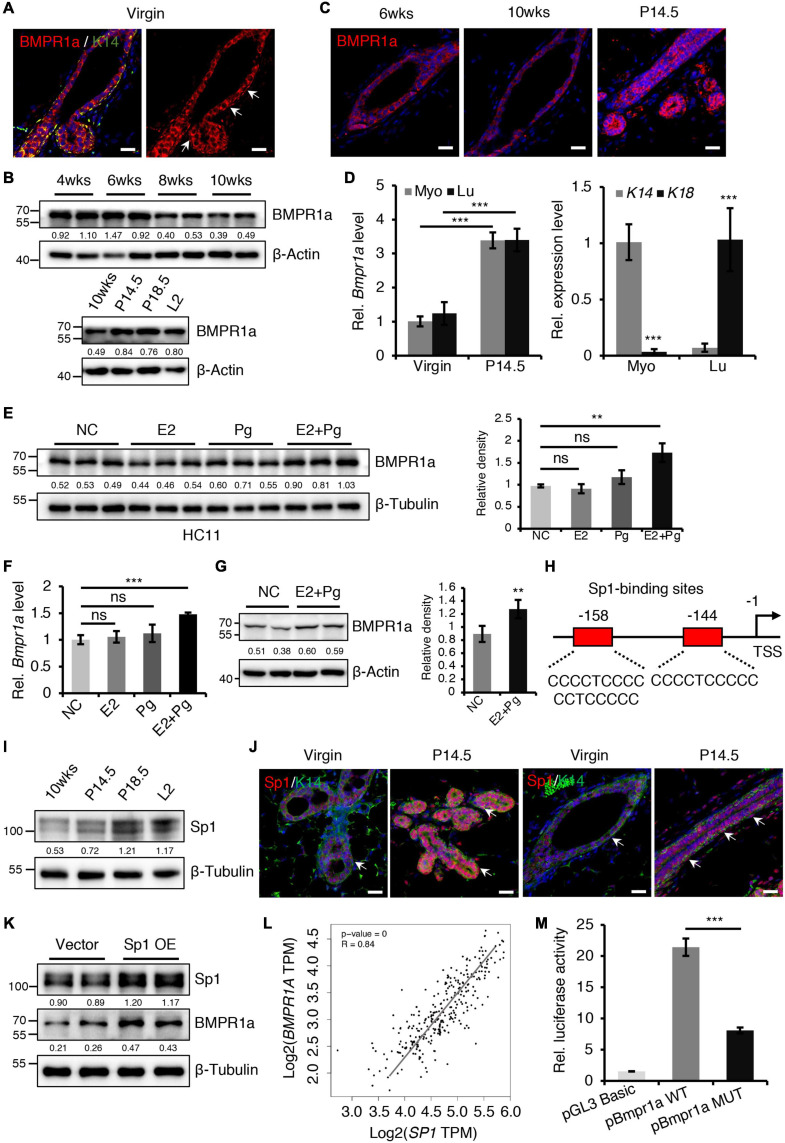
BMPR1a was upregulated by hormones during pregnancy. **(A)** Immunofluorescence staining for BMPR1a (red) and K14 (green) in 6-week-old wild-type mouse mammary gland ducts and terminal end buds. Arrows point to myoepithelial cells. *n* = 3 mice. Scale bar, 25 μm. **(B)** Western blotting for BMPR1a in isolated mammary gland epithelial cells at the indicated developmental stages (P14.5, pregnancy day 14.5; P18.5, pregnancy day 18.5; L2, lactation day 2). β-Actin was used as a loading control. **(C)** Immunofluorescence staining for BMPR1a in mammary glands of wild-type mice at the indicated time points (P14.5, pregnancy day 14.5). *n* = 3 mice per time point. Scale bar, 25 μm. **(D)** qRT-PCR analysis of *Bmpr1a* in FACS sorted myoepithelial (Myo) and luminal (Lu) cells from wild-type mice at the indicated time points (P14.5, pregnancy day 14.5) (left). qRT-PCR analysis of *K14* and *K18* in FACS-sorted myoepithelial and luminal cells from wild-type mice (right). *n* ≥ 3 biological replicates. **(E)** Western blotting for BMPR1a in HC11 cells treated with estradiol (E2, 10 nM), progesterone (Pg, 100 nM), or estradiol and progesterone (10 nM E2 + 100 nM Pg) for 24 h. Ethanol was used as negative control (NC). β-Tubulin was used as a loading control. *n* = 6 biological replicates. Statistical analysis the expression of BMPR1a/β-Tubulin. **(F)** qRT-PCR analysis of *Bmpr1a* in HC11 cells treated with estradiol (E2, 10 nM), progesterone (Pg, 100 nM), or estradiol and progesterone (10 nM E2 + 100 nM Pg) for 24 h. Ethanol was used as negative control. *n* = 3 biological replicates. **(G)** Western blotting for BMPR1a in mammary glands of ovariectomized wide-type mice with E2 and Pg stimulation for 21 days. β-Actin was used as a loading control. Statistical analysis the expression of BMPR1a/β-Actin. *n* = 4 biological replicates. **(H)** Schematic diagram showing potential Sp1-binding sites (–144/–158 bp) in the *Bmpr1a* promoter. TSS, transcription start site. **(I)** Western blotting for Sp1 in mammary epithelium at the indicated time points (P14.5, pregnancy day 14.5; P18.5, pregnancy day 18.5; L2, lactation day 2). β-Tubulin was used as a loading control. **(J)** Sp1 (red) and K14 (green) double immunofluorescence staining in the mammary gland of wild-type mice at virgin and pregnancy day 14.5 (P14.5). *n* = 3 mice per time point. Arrows point to myoepithelial cells. Scale bar, 25 μm. **(K)** Western blotting for Sp1 and BMPR1a in HC11 cells transfected with pcDNA3.1 empty vector or pcDNA3.1-Sp1 (Sp1 OE) for 24 h. β-Tubulin was used as a loading control. **(L)** Scatter plot showing the correlation between *SP1* and *BMPR1A* expression in mammary glands from TCGA and GTEx data. Pearson’s coefficient test was performed to assess statistical significance. **(M)** Luciferase activity in lysates of HC11 cells with Sp1 overexpression transfected with luciferase reporter plasmids containing the *Bmpr1a* wild-type promoter (control) or mutant promoter with mutation of Sp1-binding sites. *n* = 3 biological replicates. Data were presented as means ± SD. ^∗∗^*p* < 0.01, ^∗∗∗^*p* < 0.001. ns, not significant.

The *Bmpr1a* promoter, *p63* promoter and *Slug* promoter were cloned into pGL3-Basic reporter constructs. Sp1 binding sites of *Bmpr1a* promoter are located in NC_000080.7 34224647–34224656 34224661–34224677, Smad4 binding sites of *p63* promoter are located in NC_000082.7 25620329–25620343, Smad1 binding sites of *Slug* promoter are located in NC_000082.7 14521936–14521949, and Smad4 binding sites of *Slug* promoter are located in NC_000082.7 14523329–14523336. The nucleotide sequences of binding sites were showed in Figures 1, 5. Then Sp1-, Smad1-, or Smad4-binding sites were mutated, in which G was mutated to T and C was mutated to A, and the nucleotide sequence was verified by DNA sequencing (BGI, Beijing, China). HC11 cells were cotransfected with the phRL-TK plasmid and wild-type or mutant plasmids, respectively. Dual luciferase activity was measured after a 24 h of transfection. Dual luciferase activity assays were performed according to the Dual-Glo Luciferase Assay System (Promega) manufacturer’s instructions. The ratio of firefly luciferase to renilla luciferase was calculated.

### Chromatin Immunoprecipitation (ChIP) Assays

ChIP assays were performed using EZ-Magna ChIP G chromatin immunoprecipitation kits (17-409, Millipore) according to the manufacturer’s instructions. In brief, approximately 1 × 10^7^ cells were crosslinked with 1% formaldehyde for 10 min at room temperature. Then, the cells were resuspended in nuclear lysis buffer and sonicated cell to shear DNA into fragments of 200–1000 bp. The sonicated nuclear fractions were divided into several tubes and incubated with appropriate antibodies and protein G magnetic beads at 4°C overnight. The protein/DNA complexes were eluted and digested by Proteinase K for 2 h at 62°C with shaking, and then DNA purification was performed using spin columns. For primary mammary epithelial cells, adipocytes and red cells were first removed by enzymatic digestion, epithelial cells were collected, and cells were counted. Finally, qPCR was performed with the following primers. qPCR reaction conditions are described as follows: 10 min at 94°C followed by 50 cycles of 94°C for 20 s and 60°C for 1 min. The IP efficiency was calculated using the Percent Input Method and the equation as follows: Percent Input = 1% × 2^*Ct* (1%*InputSimple*)^ ^– Ct (IP Simple)^. The following antibodies were used: Smad1 (3 μg, 701168, Thermo Scientific), Smad4 (1 μg, ab40759, Abcam) and Normal Rabbit IgG (1 μg, 3900, CST). IgG served as a negative control. ChIP qPCR primers (5′-3′) are listed in Supplementary Table 4.

### RNA-Seq Analysis

Mammary glands were minced and digested by collagenase and hyaluronidase to remove fat tissue, and red blood cells were removed by NH_4_Cl solution. Total RNA was extracted from mammary epithelial cells which isolated from mouse mammary glands (four control and four cKO mice) at pregnancy day 14.5 and then sent to Novogene Co. Ltd. for library construction and sequencing. RNA quality was assessed using the Bioanalyzer 2100 system (Agilent Technologies, CA, United States). Sequencing libraries were generated using NEBNext UltraTM RNA Library Prep Kit for Illumina. The qualities of clean reads were assessed using FastQC (v0.11.5) and then the reads were mapped to mouse genome (GRCm38) using Hisat2 (v2.0.5). FPKM were calculated, and differential expression analysis was performed using DESeq2 (v1.16.1). Differentially expressed genes (*p* < 0.05) were chosen for further analysis. The RNA-sequencing data have been submitted to the GEO repository GSE164550.

### Statistical Analysis

Microsoft Excel software was used to perform the statistical analyses. The data are presented as the means ± SD. An unpaired two-tailed Student’s *t*-test was used to evaluate statistical significance (^∗^*p* < 0.05, ^∗∗^*p* < 0.01, ^∗∗∗^*p* < 0.001).

## Results

### BMPR1a Is Upregulated in the Mammary Epithelium During Pregnancy and Is Responsive to Hormones

To begin understanding the role of BMP signaling in the mammary gland, we first examined the expression pattern of BMP receptors in sorted myoepithelial (Lin^–^CD24^+^CD29^*high*^) and luminal (Lin^–^CD24^+^CD29^*low*^) cells from mammary glands (Supplementary Figure 1A). We found that *Bmpr1a* was predominantly expressed both in myoepithelial and luminal cells, the expression level of *Bmpr2* was moderate, *Bmpr1b* was barely detected in these subpopulations (Supplementary Figure 1B). In HC11 mouse mammary epithelial cells, the expression level of *Bmpr1a* was the highest compared with *Bmpr2* and *Bmpr1b* (Supplementary Figure 1C). Thus, we focused *Bmpr1a* in the following study. Immunofluorescence assays further revealed that BMPR1a was extensively expressed in both the myoepithelial and luminal cell layers (Figure 1A). During mammary gland development, the expression level of BMPR1a gradually decreased from puberty to adulthood, markedly increased during pregnancy (Figures 1B,C). The upregulation of *Bmpr1a* during pregnancy was further confirmed in FACS-sorted myoepithelial cells (high expression of *K14*) and luminal cells (high expression of *K18*) at the mRNA level (Figure 1D), suggesting a potential role of BMPR1a in regulating alveolar formation. To understand the niche signals that regulate BMP signaling, we have examined ligands of BMPR1a, *Bmp2* and *Bmp4*, in sorted stromal cells. qRT-PCR analysis showed that *Bmp2* and *Bmp4* were highly expressed in stromal cells, while they are barely detectable in myoepithelial and luminal cells (Supplementary Figures 1D,E). Immunohistochemistry assays further showed the specific expression of BMP4 in stromal cells of mammary gland at virginal and pregnant stages (Supplementary Figure 1F). It suggests that the BMP ligands from stromal cells activate epithelial BMP signaling in the mammary gland.

During pregnancy, mammary gland development is primarily regulated by estrogen (E2) and progesterone (Pg) (Song et al., 2019). We thus wondered whether the increase in BMPR1a expression at pregnancy is regulated by hormones. To verify this speculation, we treated HC11 mammary epithelial cells with E2 and/or Pg. We found that the expression level of BMPR1a was significantly elevated upon E2 and Pg administration, while the expression level of BMPR1a slightly but not significantly increase upon only Pg administration (Figure 1E). The results were further confirmed by qRT-PCR analysis (Figure 1F). Similarly, in ovariectomized mice, E2 and Pg induced to increase BMPR1a expression (Figure 1G). To understand how BMPR1a is regulated by E2 and Pg, we examined the *Bmpr1a* promoter using the JASPAR databases and identified potential Sp1-binding sites (Figure 1H). It is noting that Sp1 can be activated at pregnancy by E2 and Pg (Porter et al., 1997; Owen et al., 1998; Wang et al., 1998; Schultz et al., 2003; Goldhar et al., 2011). Thus, we speculated that Sp1 might regulate BMPR1a expression in response to E2 and Pg stimulation. We first validated that Sp1 was also upregulated in both the myoepithelial and luminal cell layers during pregnancy (Figures 1I,J), exhibiting a similar pattern to BMPR1a. To verify the potential regulatory effect of Sp1 on the expression of BMPR1a, we overexpressed Sp1 in HC11 mammary epithelial cells and found that Sp1 overexpression induced BMPR1a upregulation (Figure 1K). In addition, a correlation analysis showed a positive correlation between *SP1* and *BMPR1A* in normal breast (Figure 1L). Luciferase reporter assays revealed that mutation of the Sp1-binding sites blocked *Bmpr1a* promoter activity (Figure 1M). Overall, these results demonstrate that BMPR1a expression is directly regulated by Sp1, mediating E2 and Pg activity.

### Deletion of BMPR1a in the Myoepithelial Layer Results in Precocious Alveolar Differentiation

**FIGURE 2:**
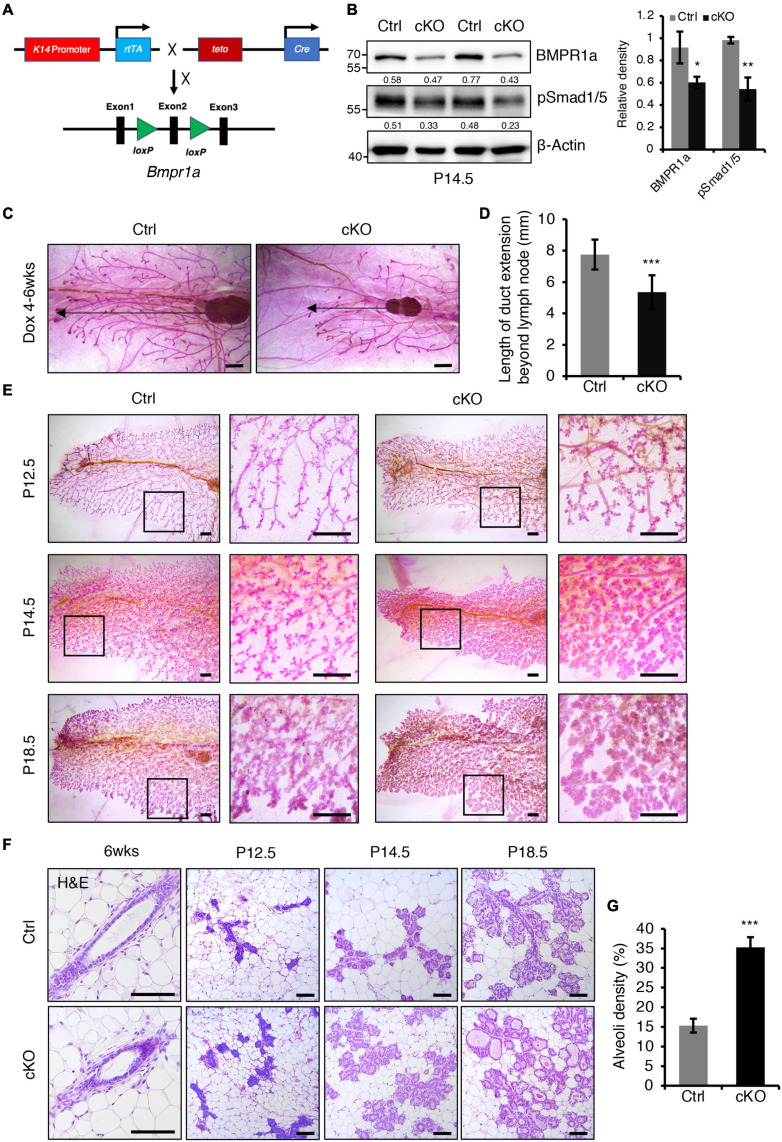
BMPR1a deficiency resulted in enlarged alveoli during pregnancy. **(A)** Schematic map of the constructs used to generate inducible mammary epithelial BMPR1a cKO mice (cKO). **(B)** Western blotting for BMPR1a and pSmad1/5 in mammary epithelial cells isolated from control (Ctrl) and cKO mice at pregnancy day 14.5 (P14.5). β-Actin was used as a loading control. Statistical analysis the expression of BMPR1a/β-Actin and pSmad1/5/β-Actin. *n* = 3 mice. **(C,D)** Whole-mount staining of mammary glands from control and cKO mice at 6 weeks of age. The mice were treated with Dox from 4 weeks of age. Quantification of duct length beyond the lymph node. *n* ≥ 5 biological replicates. Scale bar, 1 mm. **(E)** Whole-mount staining of mammary glands from control and cKO mice at pregnancy day 12.5 (P12.5) (*n* = 3 per group), pregnancy day 14.5 (P14.5) (*n* = 4 per group) and pregnancy day 18.5 (P18.5) (*n* = 3 per group). High-magnification areas are shown in black boxes. Scale bar, 1 mm. **(F)** Hematoxylin and eosin staining of mammary glands at the indicated time points (P12.5, pregnancy day 12.5; P14.5, pregnancy day 14.5; P18.5, pregnancy day 18.5). *n* = 3 mice per time point. Scale bar, 100 μm. **(G)** Quantitation of alveoli density in control and cKO mice HE-stained mammary gland sections at pregnancy day 14.5. *n* = 3 mice per group. Data were presented as means ± SD. ^∗^*p* < 0.05, ^∗∗^*p* < 0.01, ^∗∗∗^*p* < 0.001.

To investigate the *in vivo* role of BMPR1a in the mammary gland, we generated a Dox-inducible BMPR1a conditional knockout mouse model, *K14-rtTA*;*teto-Cre*;*Bmpr1a*^*fl/fl*^ mice (cKO), in which BMPR1a was inducibly deleted in mammary myoepithelial cells (Figure 2A). To validate this system, we first generated *K14-rtTA*;*teto-Cre*;*Rosa26-mTmG* mice (Supplementary Figure 2A). After Dox induction, GFP-positive cells were present in the myoepithelial cells of the duct and alveoli at P14.5 (Supplementary Figures 2B,C), corroborating the successful generation of the inducible model. BMPR1a was efficiently deleted in the mammary myoepithelium of BMPR1a cKO mice at P14.5 upon Dox induction, accompanied by downregulation of pSmad1/5 (Figure 2B). To probe its physiological function in puberty, we induced BMPR1a deletion at 4 weeks of age by oral administration of Dox-containing water. At 6 weeks of age, whole-mount analysis showed impairment of ductal elongation in BMPR1a cKO mouse mammary glands (Figures 2C,D).

Next, we examined the effect of BMPR1a loss on alveologenesis during pregnancy. Whole-mount analysis revealed that BMPR1a cKO mice formed more lobuloalveolar structures and alveolar densities than control mice at P12.5, P14.5, and P18.5 (Figures 2E,G). The mammary glands of BMPR1a cKO mice displayed expanded alveolar lumens compared with control mice at P14.5 and P18.5 (Figure 2F). These results imply that deletion of BMPR1a results in precocious alveolar differentiation at pregnancy.

**FIGURE 3:**
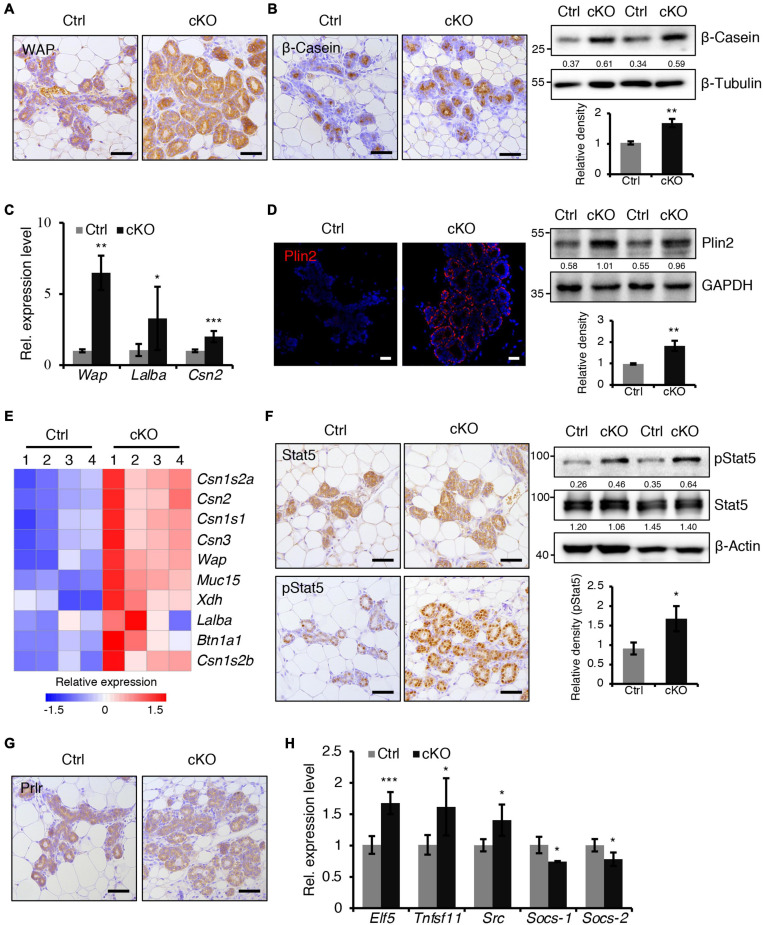
BMPR1a deficiency resulted in precocious alveolar differentiation during pregnancy. **(A)** Immunohistochemistry staining for WAP in control and cKO mammary glands at pregnancy day 14.5. *n* = 3 mice per group. Scale bar, 50 μm. **(B)** Immunohistochemistry staining and western blotting for β-Casein in control and cKO mammary glands at pregnancy day 14.5. *n* = 3 mice per group. β-Tubulin was used as a loading control. Scale bar, 50 μm. Statistical analysis the expression of β-Casein/β-Tubulin. *n* = 3 mice. **(C)** qRT-PCR analysis of *Wap*, *Lalba* and *Csn2* in mammary epithelial cells isolated from control and cKO mice at pregnancy day 14.5. *n* = 4–6 biological replicates. **(D)** Immunofluorescence staining and western blotting for Plin2 in control and cKO mammary glands at pregnancy day 14.5. *n* = 3 mice per group. GAPDH was used as a loading control. Scale bar, 25 μm. Statistical analysis the expression of Plin2/GAPDH. *n* = 3 mice. **(E)** Heatmap for milk protein-related gene expression in control and cKO mammary glands at pregnancy day 14.5. *n* = 4 mice per group. **(F)** Immunohistochemistry staining and western blotting for Stat5 and pStat5 in control and cKO mammary glands at pregnancy day 14.5. *n* = 3 mice per group. β-Actin was used as a loading control. Scale bar, 50 μm. Statistical analysis the expression of pStat5/β-Actin. *n* = 3 mice. **(G)** Immunohistochemistry staining for Prlr in control and cKO mammary glands at pregnancy day 14.5. *n* = 3 mice per group. Scale bar, 50 μm. **(H)** qRT-PCR analysis of *Elf5, Tnfsf11, Src, Socs-1*, and *Socs-2* in mammary epithelial cells isolated from control and cKO mice at pregnancy day 14.5. *n* ≥ 3 biological replicates. Data were presented as means ± SD. ^∗^*p* < 0.05, ^∗∗^*p* < 0.01, ^∗∗∗^*p* < 0.001.

At mid-pregnancy, genes encoding milk proteins such as β-Casein, Lactalbumin and Whey Acidic Protein (WAP) are initially expressed. We found that BMPR1a deficiency led to a marked upregulation of β-Casein and WAP expression at P14.5 (Figures 3A,B). The mRNA levels of *Wap*, *Lalba*, and *Csn2* were much higher in BMPR1a cKO mice than control mice (Figure 3C). In late pregnancy, mammary epithelial cells secrete cytoplasmic lipid droplets (CLDs), which are the origin of milk lipids. Plin2 is a key regulator for CLD maturation (Russell et al., 2011). A remarkable increase in Plin2 was observed in mammary alveoli of BMPR1a cKO mice at P14.5 (Figure 3D). In agreement, RNA-sequencing (RNA-seq) analysis showed that both milk synthesis-related genes, *Wap, Muc15, Csn2*, and *Lalba*, and milk lipid secretion-related genes, *Xdh* and *Btn1a1*, were markedly upregulated in BMPR1a cKO mice at P14.5 (Figure 3E).

Prlr/Stat5 signaling is critical for alveolar formation during pregnancy (Miyoshi et al., 2001). We found an increase in pStat5-positive cells in the mammary epithelium of BMPR1a cKO mice at P14.5, while Prlr expression was not altered (Figures 3F,G). Furthermore, the genes *Elf5*, *Tnfsf11*, and *Src*, which are downstream of the Prlr/Stat5 signaling pathway, were upregulated in the mammary glands of BMPR1a cKO mice, while the key attenuators *Socs-1* and *Socs-2* were downregulated (Figure 3H), suggesting an activation of the Stat5 signaling pathway. In addition, Ki67 and Cyclin D1 staining revealed fewer proliferative cells in the mammary glands of BMPR1a cKO mice than control mice at P14.5 (Supplementary Figure 3A). However, the apoptotic cells were indistinguishable between control and BMPR1a cKO mice at P18.5 (Supplementary Figure 3B). Taken together, these findings strongly indicate that BMPR1a is required to restrict precocious alveolar formation at pregnancy.

### BMPR1a Deficiency Disorders the Balance of Myoepithelial and Luminal Cells

**FIGURE 4:**
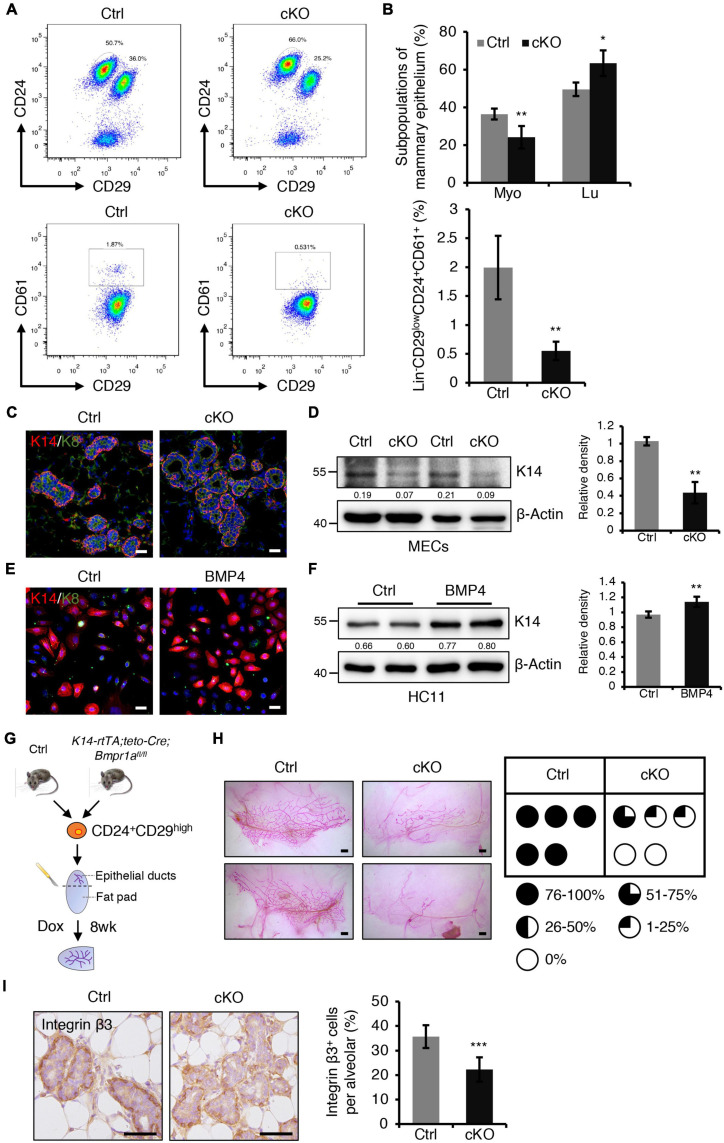
BMPR1a deficiency resulted in reduced myoepithelial lineages and compromised repopulating capacity of mammary stem cells. **(A)** FACS analysis of mammary gland epithelial cells from control and cKO mice at pregnancy day 14.5 using the Lin/CD24/CD29/CD61 surface marker set. *n* = 4 mice per group. **(B)** Quantification of Lin^–^ CD24^+^CD29^*high*^ (Myo), Lin^–^ CD24^+^CD29^*low*^ (Lu) and Lin^–^ CD24^+^CD29^*low*^CD61^+^ cell populations in control and cKO mice at pregnancy day 14.5. *n* = 4 biological replicates. **(C)** Immunofluorescence staining for K14 (red) and K8 (green) in control and cKO mammary glands at pregnancy day 14.5. *n* = 3 mice per group. Scale bar, 25 μm. **(D)** Western blotting for K14 in control and cKO mammary glands at pregnancy day 14.5. β-Actin was used as a loading control. Statistical analysis the expression of K14/β-Actin. *n* = 3 mice. **(E)** Immunofluorescence staining for K14 (red) and K8 (green) in HC11 mammary epithelial cells treated with BMP4 (50 ng/mL) for 24 h. *n* = 3 biological replicates. Scale bar, 25 μm. **(F)** Western blotting for K14 in HC11 cells treated with BMP4 (50 ng/mL) for 24 h. β-Actin was used as a loading control. Statistical analysis the expression of K14/β-Actin. *n* = 4 biological replicates. **(G)** Illustration of the mammary transplantation assays using *K14-rtTA/teto-Cre/Bmpr1a^*fl/fl*^* and control mouse mammary stem cells and the induction strategy. **(H)** Whole-mount staining of mammary glands from the mammary transplantation assays and quantification of the proportion of ductal trees. *n* = 5 mice per group. Scale bar, 1 mm. **(I)** Immunohistochemistry staining for Integrin β3 in control and cKO mammary glands at pregnancy day 14.5. Quantification of Integrin β3^+^ cells in alveoli. *n* = 3 mice per group. Scale bar, 50 μm. Data were presented as means ± SD. ^∗^*p* < 0.05, ^∗∗^*p* < 0.01, ^∗∗∗^*p* < 0.001.

The balance of myoepithelial and luminal cells is important for alveolar development. We next sought to examine the effect of BMPR1a deficiency on this balance. Fluorescence activated cell sorting (FACS) revealed a reduction in the Lin^–^CD24^+^CD29^*high*^ myoepithelial cell population and an increase in the Lin^–^CD24^+^CD29^*low*^ luminal cell population in BMPR1a cKO mice at P14.5 (Figures 4A,B). CD61 is a marker of the luminal/alveolar progenitor cell pool (Asselin-Labat et al., 2007). We observed a marked reduction in the Lin^–^CD24^+^CD29^*low*^CD61^+^ cell population in BMPR1a cKO mice (Figures 4A,B). The data suggest that BMPR1a deficiency disorders the balance of myoepithelial and luminal cells and promotes alveolar maturation.

Furthermore, dual immunofluorescence staining for K14 and K8 revealed that K14^+^ myoepithelial cells were reduced in BMPR1a cKO mice (Figure 4C). Consistently, western blotting demonstrated a reduction in K14 in the BMPR1a cKO mammary epithelium (Figure 4D). To test whether BMP signaling directly modulates myoepithelial cells, we activated BMP signaling in HC11 mammary epithelial cells by adding BMP2 or BMP4, and activation of BMP signaling was confirmed by upregulation of the BMP target genes *Id1*, *Id2*, *Id3*, and *Msx1* (Supplementary Figures 4A,B). To prove that BMPR1a mediates the BMP2- or BMP4-induced BMP signaling activation, we knockdown *Bmpr1a* in HC11 cells, then treated the cells with BMP2 or BMP4. *Bmpr1a* inhibition dampened the BMP signaling activation (Supplementary Figures 4A,B). Activated BMP signaling led to an increase in K14^+^ cells, as well as upregulation of K14 protein (Figure 4E,F). In contrast, the expression of myoepithelial marker K14 was significantly reduced in *Bmpr1a*-knockdown cells upon BMP4 stimulation (Supplementary Figure 5A). These findings suggest that the activation of BMP signaling is critical for maintaining the fate of myoepithelial cells. Furthermore, in order to test whether BMP signaling mediated the function of estrogen and progesterone hormones, HC11 cells were stimulated with E2 + Pg and then treated with/without BMP4. qRT-PCR analysis showed that the expression level of *K14* is higher upon E2 + Pg stimulation and BMP4 treatment than upon only BMP4 treatment (Supplementary Figure 5B). It implies that estrogen- and progesterone-induced BMPR1a upregulation is important to maintain mammary epithelial cells in a myoepithelial state.

Mammary stem cells are enriched in the CD24^+^CD29^*high*^ cell population, and these stem cells can repopulate the mammary gland after transplantation. To further test whether BMPR1a deficiency impairs the repopulating capacity of mammary stem cells, we sorted Lin^–^CD24^+^CD29^*high*^ cells from control and *K14-rtTA;teto-Cre;Bmpr1a^*fl/fl*^* mouse mammary glands and transplanted them into the cleared fat pad of immunodeficient mice. After transplantation, Dox was orally administered to the mice immediately and last for eight weeks (Figure 4G). We found that BMPR1a deficiency resulted in compromised mammary outgrowth (Figure 4H), suggesting an impairment of repopulating capacity. It has been reported that the BMP pathway target genes *Id1, Id2, Id3, Msx1*, and *Msx2* regulate stemness and differentiation in many types of cells (Ge et al., 2019; Wang et al., 2019). The expression levels of *Id2*, *Id3*, *Msx1*, and *Msx2* were downregulated in BMPR1a cKO mouse mammary myoepithelial cells (Supplementary Figure 4C). It has been reported that Integrin β3 marks mammary stem cells and luminal progenitor cells in the mammary gland (Desgrosellier et al., 2014). We found that the number of Integrin β3^+^ cells significantly reduced in the mammary gland from BMPR1a cKO mice, especially in the myoepithelial layer (Figure 4I), suggesting that BMPR1a deficiency could impair repopulating capacity.

Taken together, these data indicate that BMPR1a is important for maintaining myoepithelial cell identity and fate determinant.

### BMPR1a Regulates p63 and Slug Expression Through pSmad1-Smad4 Complexes in the Mammary Gland

**FIGURE 5:**
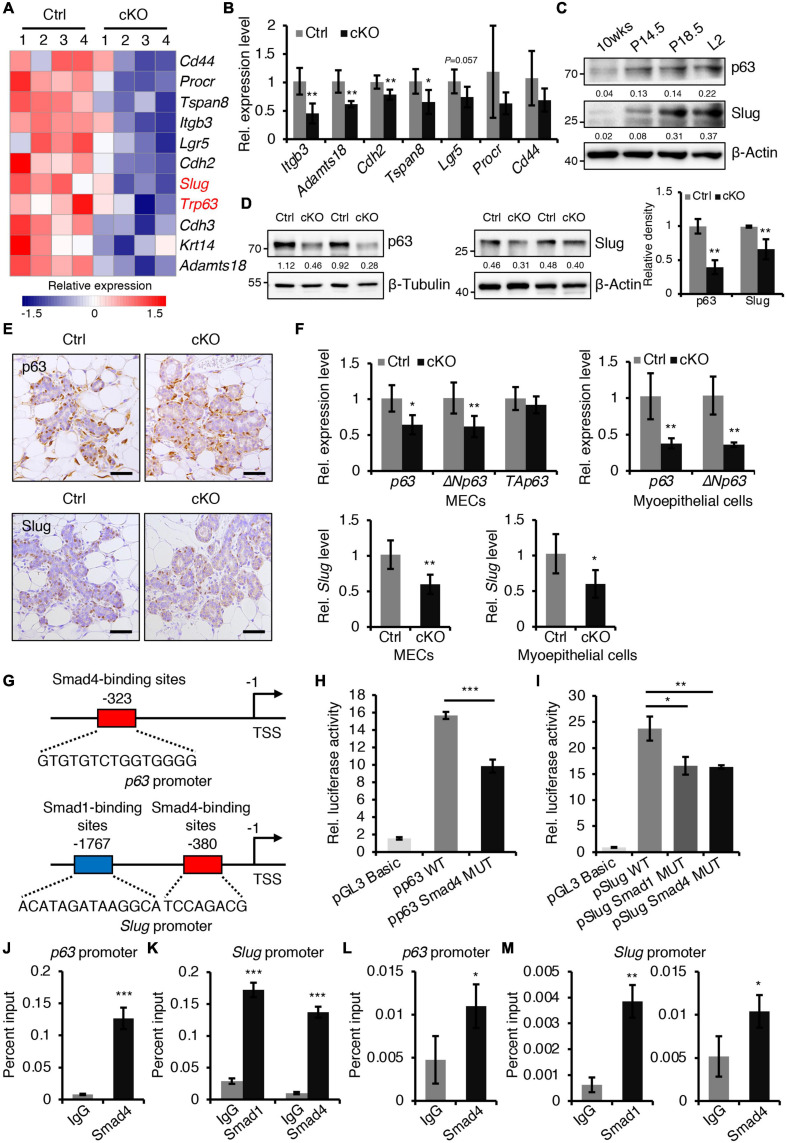
BMPR1a regulated the expression of p63 and Slug through the pSmad1/5-Smad4 complex. **(A)** Heatmap for myoepithelial layer-related gene expression in control and cKO mammary glands at pregnancy day 14.5. *n* = 4 mice per group. **(B)** qRT-PCR analysis of *Itgb3, Adamts18, Cdh2, Tspan8, Lgr5, Procr*, and *Cd44* in FACS-sorted myoepithelial cells from control and cKO mice at pregnancy day 14.5. *n* = 4–5 biological replicates. **(C)** Western blotting for p63 and Slug in wild-type mouse mammary glands at the indicated time points (P14.5, pregnancy day 14.5; P18.5, pregnancy day 18.5; L2, lactation day 2). β-Actin was used as a loading control. **(D)** Western blotting for p63 and Slug in mammary epithelial cells isolated from control and cKO mice at pregnancy day 14.5. β-Tubulin and β-Actin were used as loading controls. Statistical analysis the expression of p63/β-Tubulin (*n* = 3 mice) and Slug/β-Actin (*n* = 4 mice). **(E)** Immunohistochemistry staining for p63 and Slug in control and cKO mammary glands at pregnancy day 14.5. *n* = 4 mice per group. Scale bar, 50 μm. **(F)** qRT-PCR analysis of *p63*,Δ*Np63, TAp63*, and *Slug* in isolated mammary epithelial cells (MECs) and FACS-sorted myoepithelial cells from control and cKO mice at pregnancy day 14.5. *n* = 4-6 biological replicates. **(G)** Schematic diagram showing potential Smad4-binding sites (–323 bp) in the *p63* promoter and potential Smad1-binding sites (–1767 bp) and Smad4-binding sites (–380 bp) in the *Slug* promoter. **(H)** Luciferase activity in lysates of HC11 cells transfected with pGL3-basic empty vector, wild-type *p63* promoter (control), or mutant promoter (with mutation of Smad4-binding sites) luciferase reporter plasmids. *n* = 3 biological replicates. **(I)** Luciferase activity in lysates of HC11 cells transfected with pGL3-basic empty vector, wild-type *Slug* promoter (control), or mutant promoter (with mutation of Smad1- or Smad4-binding sites) luciferase reporter plasmids. *n* = 3 biological replicates. **(J)** ChIP analysis for the binding sites of Smad4 in the *p63* promoter in HC11 mammary epithelial cells treated with BMP4 for 1 h using antibodies against Smad4. IgG was used as a negative control. The enrichment of Smad4 binding to *p63* promoter was quantified using qPCR. *n* = 3. **(K)** ChIP analysis for the binding sites of Smad1 or Smad4 in the *Slug* promoter in HC11 mammary epithelial cells treated with BMP4 for 1 h using antibodies against Smad1 or Smad4. IgG was used as a negative control. The enrichment of Smad1 or Smad4 binding to *Slug* promoter was quantified using qPCR. *n* = 3. **(L)** ChIP analysis for the binding sites of Smad4 in the *p63* promoter in primary mammary epithelial cells isolated from mouse mammary glands at pregnancy day 14.5 using antibodies against Smad4. IgG was used as a negative control. The enrichment of Smad4 binding to *p63* promoter was quantified using qPCR. *n* = 3. **(M)** ChIP analysis for the binding sites of Smad1 or Smad4 in the *Slug* promoter in primary mammary epithelial cells isolated from mouse mammary glands at pregnancy day 14.5 using antibodies against Smad1 or Smad4. IgG was used as a negative control. The enrichment of Smad1 or Smad4 binding to *Slug* promoter was quantified using qPCR. *n* = 3. Data were presented as means ± SD. ^∗^*p* < 0.05, ^∗∗^*p* < 0.01, ^∗∗∗^*p* < 0.001.

To gain insight into the molecular mechanisms of BMP signaling in mammary development, RNA-seq was performed on mammary epithelium isolated from control and BMPR1a cKO mice at P14.5. The differentially expressed gene analysis indicated that loss of BMPR1a led to a reduction in most myoepithelial layer-associated genes (Figure 5A). qRT-PCR data showed that the expression levels of *Itgb3, Adamts18, Cdh2*, and *Tspan8* were significantly downregulated in FACS-sorted myoepithelial cells (Figure 5B). Interestingly, *p63* and *Slug*, which are key regulators of myoepithelial cell fate (Crum and McKeon, 2010; Phillips and Kuperwasser, 2014), were also downregulated in BMPR1a cKO mice (Figure 5A). The reduction in p63 and Slug was validated at the protein level (Figure 5D). In line with this, p63- and Slug-positive cells were markedly reduced in alveoli from BMPR1a cKO mice (Figure 5E). In addition, the mRNA levels of *p63*, the *p63* isoform Δ*Np63*, and *Slug* were reduced in BMPR1a cKO mammary epithelium at P14.5, and the reduction was more pronounced in myoepithelial cells (Figure 5F). The expression levels of p63 and Slug were significantly upregulated in the mammary epithelium after pregnancy (Figure 5C), which is consistent with the expression pattern of BMPR1a. A correlation analysis revealed a positive correlation between *BMPR1A* and *P63* or between *BMPR1A* and *SLUG* in the normal mammary gland (Supplementary Figure 6A). This suggests that *p63* and *Slug* may be regulated by BMP signaling.

To confirm the regulatory effects *in vitro*, we activated BMP signaling in HC11 mammary epithelial cells with BMP4. The activity of pSmad1/5 increased upon BMP4 treatment (Supplementary Figures 6B,C). As expected, activated BMP signaling resulted in an increase in p63 and Slug (Supplementary Figures 6D,E). Similarly, BMP2 stimulation also upregulated the expression levels of Slug and p63 (Supplementary Figure 6F) and myoepithelial marker K14 (Supplementary Figures 6G,H) in HC11 cells. Therefore, we speculated that *p63* and *Slug* may be downstream genes of BMP signaling in the mammary gland. To prove this, we examined the *p63* and *Slug* promoters using the JASPAR databases. The analysis identified Smad4-binding sites in the *p63* promoter and Smad1- and Smad4-binding sites in the *Slug* promoter (Figure 5G). Luciferase reporter assays revealed that mutation of the Smad4-binding sites decreased *p63* promoter activity, and mutation of the Smad1- or Smad4-binding sites decreased *Slug* promoter activity respectively (Figures 5H,I). Furthermore, ChIP assays indicated that Smad1 binds its sites on the *Slug* promoter and Smad4 binds its sites on the *p63* or *Slug* promoter both in HC11 mammary epithelial cells and in primary mammary epithelial cells at P14.5 (Figures 5J–M).

Next, to confirm that *p63* and *Slug* function as downstream target genes of BMP signaling in the mammary gland, we knocked down *p63* or *Slug* in HC11 mammary epithelial cells. Suppression of *p63* or *Slug* decreased the expression of the myoepithelial marker K14 and increased the expression of the luminal marker K8/K18 (Supplementary Figures 7A–D). Immunostaining assays showed that K14^+^ cells were reduced upon suppression of *p63* or *Slug*, whereas K8^+^ cells were increased (Supplementary Figures 7E,F).

Collectively, these results demonstrate that activated BMP signaling directly regulates the expression of *p63* and *Slug* through the pSmad1/5-Smad4 complex, consequently maintaining the balance of myoepithelial and luminal lineages.

### BMPR1a Prevents Alveolar Epithelial Precocity by Regulating P-Cadherin and Spp1

**FIGURE 6:**
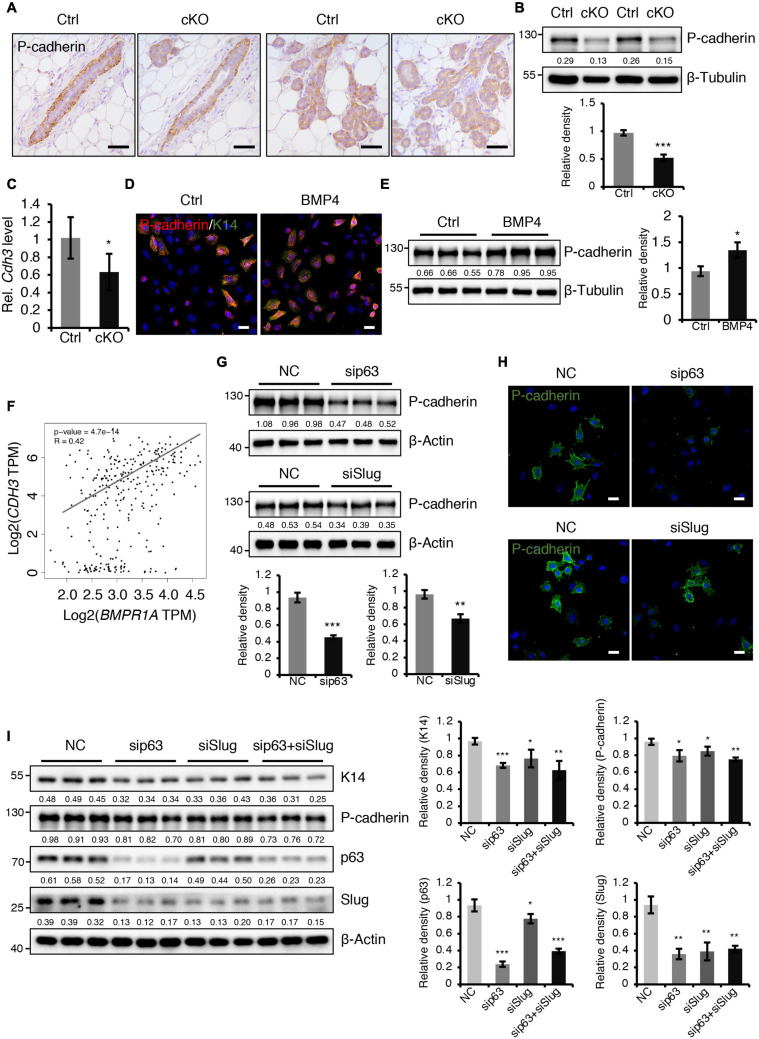
BMPR1a regulated P-cadherin expression via p63 and Slug. **(A)** Immunohistochemistry staining for P-cadherin in control (*n* = 4 mice) and cKO (*n* = 3 mice) mammary glands at pregnancy day 14.5. Scale bar, 50 μm. **(B)** Western blotting for P-cadherin in mammary epithelial cells isolated from control and cKO mice at pregnancy day 14.5. β-Tubulin was used as a loading control. Statistical analysis the expression of P-cadherin/β-Tubulin. *n* = 3 mice. **(C)** qRT-PCR analysis of *Cdh3* in FACS-sorted control and cKO myoepithelial cells at pregnancy day 14.5. *n* = 4 biological replicates. **(D)** P-cadherin (red) and K14 (green) double immunofluorescence staining in HC11 mammary epithelial cells treated with BMP4 (50 ng/mL) for 24 h. *n* = 3 biological replicates. Scale bar, 25 μm. **(E)** Western blotting for P-cadherin in HC11 mammary epithelial cells treated with BMP4 (50 ng/mL) for 24 h. β-Tubulin was used as a loading control. Statistical analysis the expression of P-cadherin/β-Tubulin. *n* = 3 biological replicates. **(F)** Scatter plot showing the correlation between *BMPR1A* and *CDH3* expression in mammary glands from TCGA and GTEx data. Pearson’s coefficient test was performed to assess statistical significance. **(G)** Western blotting for P-cadherin in HC11 cells treated with p63 siRNA (sip63)/Slug siRNA (siSlug) and scramble RNA (NC) at 48 h. β-Actin was used as a loading control. Statistical analysis the expression of P-cadherin/β-Actin. *n* = 3 biological replicates. **(H)** Immunofluorescence for P-cadherin (green) in HC11 cells treated with p63 siRNA (sip63)/Slug siRNA (siSlug) and scramble RNA (NC) at 48 h. *n* = 3 biological replicates. Scale bar, 25 μm. **(I)** Western blotting for K14, P-cadherin, p63 and Slug in HC11 cells treated with scramble RNA, p63 siRNA, Slug siRNA, or p63 siRNA and Slug siRNA at 48 h. β-Actin was used as a loading control. Statistical analysis the expression of K14/β-Actin, P-cadherin/β-Actin, p63/β-Actin and Slug/β-Actin. *n* = 3 biological replicates. Data were presented as means ± SD. ^∗^*p* < 0.05, ^∗∗^*p* < 0.01, ^∗∗∗^*p* < 0.001.

A previously published study demonstrated that loss of P-cadherin, a classical cell-to-cell adhesion molecule that is exclusively expressed in myoepithelial cells of the mammary gland (Albergaria et al., 2011), leads to precocious alveolar differentiation phenotype (Radice et al., 1997), which is similar to the phenotype seen in BMPR1a cKO mice. We thus wondered whether P-cadherin is regulated by BMP signaling. We observed that the protein and mRNA levels of P-cadherin were markedly reduced in the mammary epithelium of BMPR1a cKO mice at P14.5 (Figures 6A–C). To further test the regulatory effect of BMP signaling on P-cadherin, we treated HC11 mammary epithelial cells with BMP4 to activate BMP signaling. We found an upregulation of P-cadherin in response to BMP4 treatment (Figures 6D,E), suggesting a direct effect of BMP signaling on P-cadherin. Consistently, a correlation analysis in normal breast showed a positive correlation between *BMPR1A* and *CDH3* (gene symbol of P-cadherin) (Figure 6F). It has been reported that *Cdh3* is a p63 target gene in limb buds and hair follicles, and that Slug can also bind and activate the *Cdh3* promoter in CommaDβ cells (Shimomura et al., 2008; Idoux-Gillet et al., 2018). Thus, we wondered whether P-cadherin can be regulated by p63 and Slug in mammary epithelial cells. We found that inhibition of p63 or Slug led to a decrease in the expression of P-cadherin (Figures 6G,H), suggesting a regulatory effect of p63 or Slug on P-cadherin in the mammary gland. Moreover, suppression of both p63 and Slug resulted in a more robust downregulation of K14 and P-cadherin than only p63 or only Slug (Figure 6I). It is worth noting that the expression level of Slug was dramatically downregulated upon knockdown of p63 (Figure 6I), suggesting that Slug could be a downstream effector of p63. Considering the above findings that p63 and Slug are downstream target genes of BMPR1a, it suggests that BMP signaling restricts alveolar precocity via the BMPR1a/p63/P-cadherin and BMPR1a/Slug/P-cadherin pathways.

**FIGURE 7:**
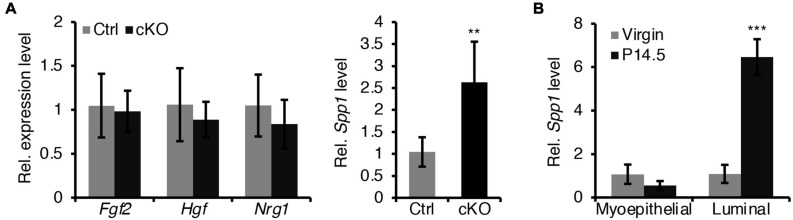
Loss of BMPR1a in myoepithelium resulted in an upregulation of *Spp1.* **(A)** qRT-PCR analysis of *Fgf2*, *Hgf*, *Nrg1*, and *Spp1* in sorted mammary myoepithelial cells from control and cKO mice at pregnancy day 14.5. *n* ≥ 4 biological replicates. **(B)** qRT-PCR analysis of *Spp1* in sorted myoepithelial and luminal cells from wild-type mice at virginal stage and pregnancy day 14.5 (P14.5). *n* ≥ 3 biological replicates. Data were presented as means ± SD. ^∗∗^*p* < 0.01, ^∗∗∗^*p* < 0.001.

In addition, we sought to test whether a defective paracrine basal-to-luminal cell signaling could result in the precocious alveolar differentiation. To test this idea, we have examined several genes expression of secreted proteins including *Spp1*, *Nrg1*, *Hgf*, and *Fgf2* in myoepithelial cells (Figure 7A). Interestingly, we did find that *Spp1*, which encodes a secreted protein, was markedly upregulated in myoepithelial cells in BMPR1a cKO mice (Figure 7A) and it was markedly upregulated in luminal cells during pregnancy (Figure 7B). It has been reported that overexpression of *Spp1* led to alveolar formation (Hubbard et al., 2013). Thus, the upregulation of *Spp1* could account for the precocious alveolar differentiation phenotype.

## Discussion

**FIGURE 8:**
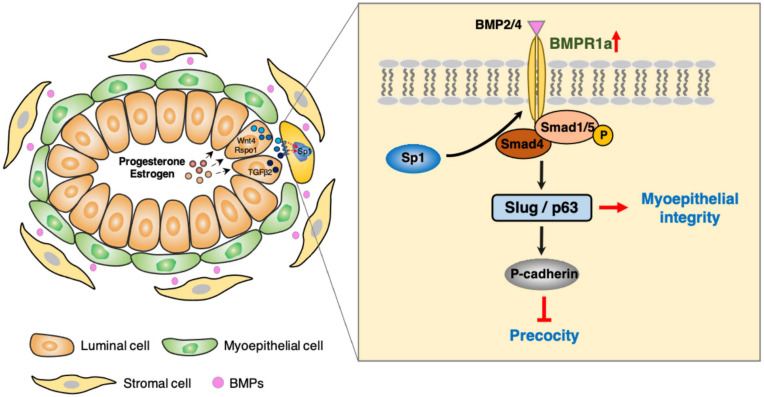
Working model of the role of BMPR1a in regulating mammary myoepithelial cells.

In this study, we found that BMPR1a is upregulated by hormones at pregnancy, and BMPR1a regulates *p63* and *Slug* to promote expansion of the myoepithelial lineage and the repopulating capacity of mammary stem cells, and to restrict alveolar precocity, identifying a novel physiological role of BMP signaling in the mammary gland (Figure 8). It is interesting that BMPR1a is directly regulated by Sp1, mediating estrogen and progesterone activity. During pregnancy, progesterone induces PR^+^ cells to secrete TGFβ2, Wnt4, and Rspo1. Previous studies showed that TGFβ2 can upregulate Sp1 expression in myoepithelial cells (Desgrosellier et al., 2014). In addition, Wnt4- and Rspo1-mediated Wnt activation can stabilize Sp1 protein (Cai et al., 2014; Rajaram et al., 2015; Mir et al., 2018; Ataca et al., 2020). Given this, Sp1 mediates the regulation of estrogen and progesterone on BMPR1a in myoepithelial cells during pregnancy. Considering the importance of BMPR1a in determining myoepithelial cell fate and maintaining mammary stem cell activity, we proposed a model in which upon luminal hormone signal induced BMPR1a upregulation maintains myoepithelial cells in an undifferentiated state during pregnancy.

During pregnancy, alveolar epithelial cells largely expand in a stem cell-driving manner (Rios et al., 2014). The myoepithelial/basal cell layer is thought to be enriched with mammary stem cells and increases mammary stem cell activity during pregnancy (Asselin-Labat et al., 2010). Here, we observed that deletion of BMPR1a in myoepithelial cells resulted in a reduction in the CD24^+^CD29^*high*^ cell population and compromised repopulating capacity, which are similar to those observed in the mammary glands of p63 or Slug KO mice (Chakrabarti et al., 2014; Phillips et al., 2014). Previous studies have demonstrated that p63 and Slug maintain mammary stem cell activity and determine myoepithelial cell fate (Guo et al., 2012; Chakrabarti et al., 2014; Phillips et al., 2014). In line with these previous studies, our findings indicate that BMPR1a regulates p63 and Slug expression, which sustain mammary stem cells and myoepithelial integrity. In support of this notion, BMP4 promotes stem cell activity in MCF10A human mammary epithelial cells (Choi et al., 2019), while inhibition of BMP signaling leads to a reduction in the ALDH1^+^ stem cell population in HC11 mammary epithelial cells (Balboni et al., 2013). Thus, it appears that BMP signaling is important for maintaining mammary stem cell activity. A recent study showed that increased BMP signaling is involved in the conversion of luminal cells to luminal-derived basal cells (LdBCs) (Song et al., 2019) further supported the importance of BMP signaling in maintaining stemness of mammary stem cells. In addition to the influence of BMP signaling on mammary stem cells, we observed that deletion of BMPR1a in myoepithelial cells can cause a defective basal-to-luminal paracrine signaling, resulting in defective luminal cell differentiation. Thus, loss of BMPR1a could impair both the repopulating capacity of mammary stem cells and the differentiation of mammary epithelial cells derived from basal epithelial cells.

Mechanistically, BMP signaling regulates p63 and Slug expression via Smad4 and/or Smad1. It is worth noting that Smad1 is a specific transcription factor for BMP signaling, while Smad4 is a common regulator that mediates signaling pathways of BMP, activin and TGFβ (Shi and Massagué, 2003). It appears that the BMP, activin and TGFβ signaling pathways could interplay to regulate the expression of p63 and Slug, consequently governing mammary gland development and lobuloalveolar formation. It merits further investigation on the function of activin and TGFβ signaling pathways in myoepithelial cell lineage.

Another interesting finding is that BMPR1a loss in myoepithelial cells results in precocious alveolar differentiation and an enlarged lumen of alveoli at pregnancy. These findings demonstrate that myoepithelial BMP signaling appropriately sustains the luminal development process. The expression of myoepithelial P-cadherin is vital for maintaining the undifferentiated state of luminal cells to prevent precocity (Radice et al., 1997; Albergaria et al., 2011), and a reduction in P-cadherin expression was observed in BMPR1a cKO mice. In this study, we identified two downstream target genes of BMP signaling, p63 and Slug, which can induce P-cadherin expression (Shimomura et al., 2008; Idoux-Gillet et al., 2018). Thus, it appears that elevated BMPR1a prevents alveolar precocity via the BMPR1a/p63/P-cadherin and BMPR1a/Slug/*P*-cadherin pathways. In addition, the upregulation of Spp1 in myoepithelial cells of BMPR1a cKO mice could account for the precocious alveolar differentiation. In line with our findings, previous studies have shown that Smad4 and Slug inhibit the expression of *Spp1* in mice (Ding et al., 2011; Wei et al., 2020) and that Spp1 contributes to alveologenesis (Hubbard et al., 2013). It implies that Spp1 might be a downstream regulator of BMP signaling that can be secreted outside of myoepithelial cells and then promotes precocious alveolar formation.

## Data Availability Statement

The datasets presented in this study can be found in online repositories. The names of the repository/repositories and accession number(s) can be found below: https://www.ncbi.nlm.nih.gov/geo/, GSE164550.

## Ethics Statement

The animal study was reviewed and approved by the Regulations of Beijing Laboratory Animal Management and the Institutional Animal Care and Use Committee of China Agricultural University (SKLAB-2015-01-03).

## Author Contributions

ZY and CL designed the experiments. CS, PL, RL, XB, GL, XY, XS, and JX performed the experiments. CS and PL analyzed the data. CS and ZY wrote the manuscript. All the authors contributed to the article and approved the submitted version.

## Conflict of Interest

The authors declare that the research was conducted in the absence of any commercial or financial relationships that could be construed as a potential conflict of interest.
