# Supplementary material for: Hormone-Responsive BMP Signaling Expands Myoepithelial Cell Lineages and Prevents Alveolar Precocity in Mammary Gland
Source: Front Cell Dev Biol. 2021 Jul 15;9:691050. doi: 10.3389/fcell.2021.691050 (PMC8320003; doi:10.3389/fcell.2021.691050)
Supplement: Supplementary file 1 [file Data_Sheet_1.PDF]

Supplementary materials

The supplementary materials include 4 supplementary tables and 7 supplementary Figures and Legends.

Supplementary Figures :

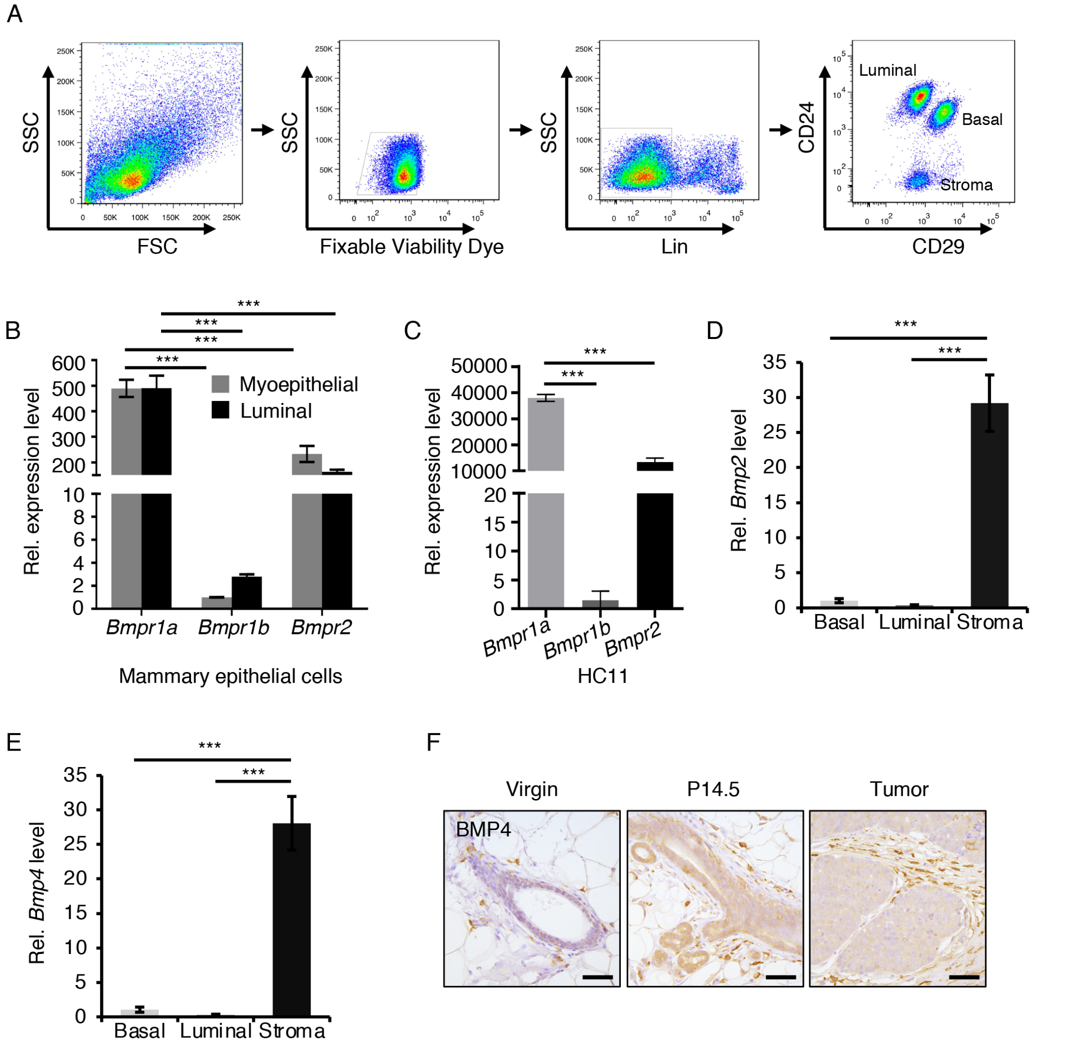

Figure S1. The expression pattern of different BMP receptors and ligands in the mammary gland

(A) Strategy for sorting myoepithelial, luminal and stromal cells from the mammary gland. Lin: CD45, CD31 and TER119. **(B)** qRT-PCR analysis of *Bmpr1a*, *Bmpr1b* and *Bmpr2* in sorted myoepithelial and luminal cells from wild-type mouse mammary glands at pregnancy day 14.5. n = 3-4 biological replicates. **(C)** qRT-PCR analysis of *Bmpr1a*, *Bmpr1b* and *Bmpr2* in HC11 mouse mammary epithelial cells. n = 3 biological replicates. **(D)** qRT-PCR analysis of *Bmp2* in sorted basal, luminal and stromal cells from wild-type mouse mammary glands at 10 weeks of age. n = 4 biological replicates. **(E)** qRT-PCR analysis of *Bmp4* in sorted basal, luminal and stromal cells from wild-type mouse mammary glands at 10 weeks of age. n = 3 biological replicates. **(F)** Immunohistochemistry staining for BMP4 in the mammary gland at virgin (n = 3) and pregnancy day 14.5 (P14.5) (n = 3). *PyVT* tumor section was used as a positive control. Scale bar, 50 µm. Data were presented as means ± SD. $^{***}p < 0.001$.

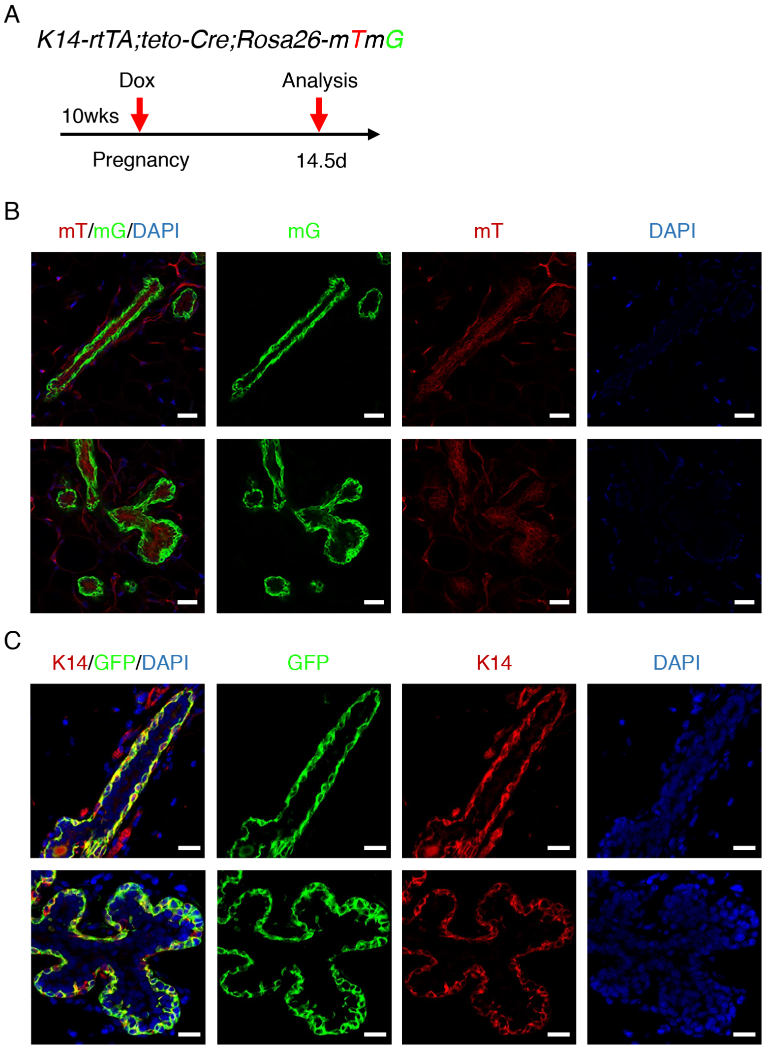

Figure S2. Validation of the efficiency of Cre recombinase in mammary gland in the Dox inducible system using *K14-rtTA;teto-Cre;Rosa26-mTmG* mice

(A) Strategy for Dox induction in *K14-rtTA;teto-Cre;mTmG* mice during pregnancy. **(B)** Confocal imaging of mT (red) and mG (green) in the mammary gland at pregnancy day 14.5. n = 3 mice. Scale bar, 25 µm. **(C)** Immunofluorescence staining for K14 (red) and GFP (green) in mammary glands from *K14-rtTA;teto-Cre;mTmG* mice at pregnancy day 14.5. n = 3 mice. Scale bar, 25 µm.

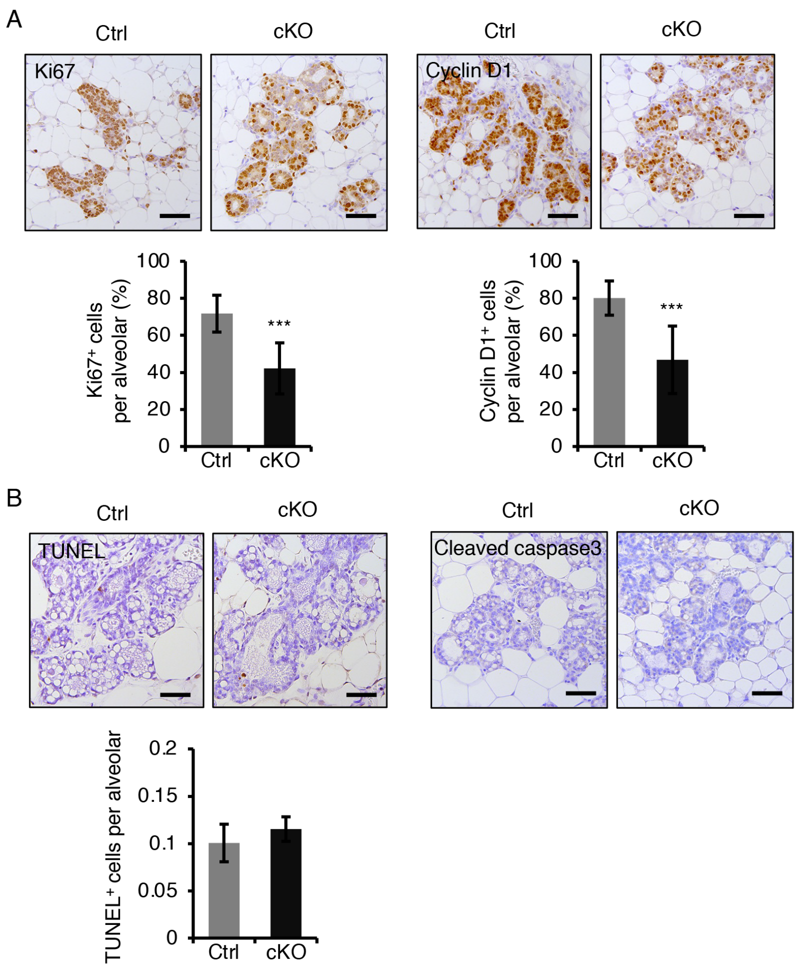

Figure S3. Deletion of BMPR1a in the myoepithelium suppressed cell proliferation in the mammary epithelium

(A) Immunohistochemistry staining for Ki67 and Cyclin D1 in control and cKO mammary glands at pregnancy day 14.5 and quantification of $Ki67^{+}$ and Cyclin $D1^{+}$ cells in alveoli. n = 3 mice per group. Scale bar, 50 μm. **(B)** TUNEL staining and immunohistochemistry staining for Cleaved caspase3 in control and cKO mammary glands at pregnancy day 18.5. Quantification of $TUNEL^{+}$ cells in per alveoli. n = 3 mice per group. Scale bar, 50 μm. Data were presented as means ± SD. $^{***}p < 0.001$.

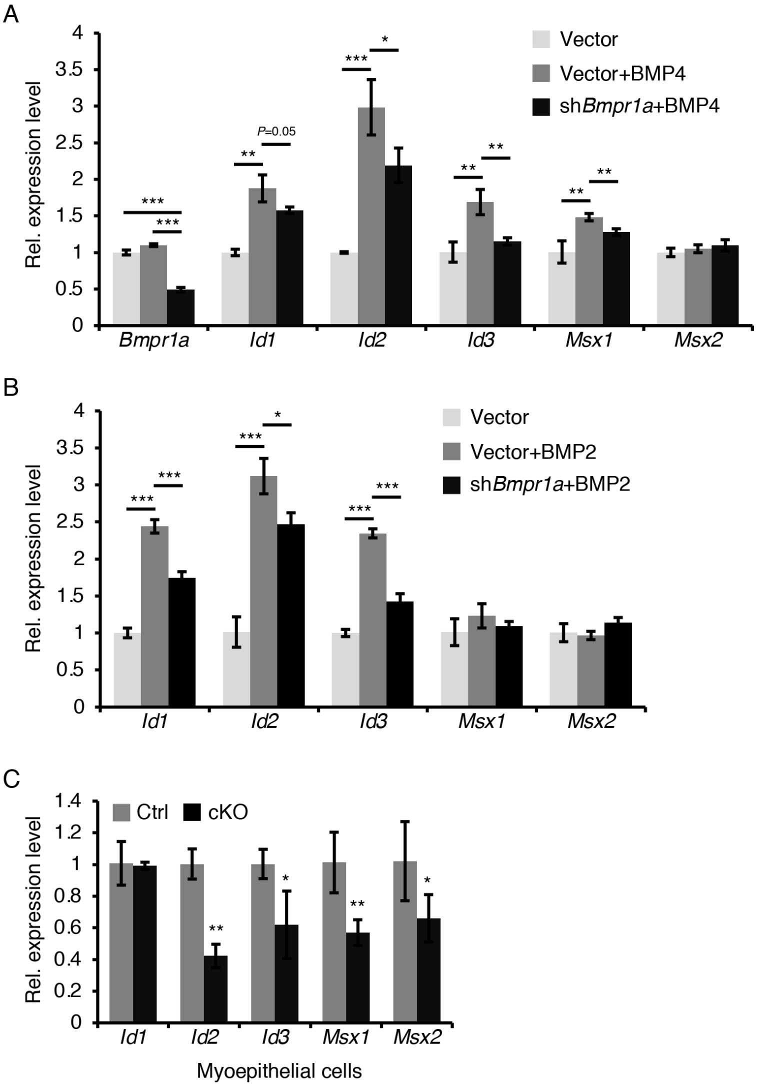

Figure S4. Deletion of BMPR1a led to a reduction in the target genes of BMP signaling pathway in myoepithelial cells

(A) Knockdown *Bmpr1a* expression in HC11 cells by shRNA, and then treated the control (Vector) and *Bmpr1a*-knockdown (sh*Bmpr1a*) cells with BMP4 (50 ng/mL) for 24 h. qRT-PCR analysis of *Bmpr1a, Id1, Id2, Id3, Msx1* and *Msx2* in the HC11 cells under indicated conditions. n = 3 biological replicates. **(B)** Knockdown *Bmpr1a* expression in HC11 cells by shRNA, and then treated the control (Vector) and *Bmpr1a*-knockdown (sh*Bmpr1a*) cells with BMP2 (50 ng/mL) for 24 h. qRT-PCR analysis of *Id1, Id2, Id3, Msx1* and *Msx2* in the HC11 cells under indicated conditions. n = 3 biological replicates. **(C)** qRT-PCR analysis of *Id1, Id2, Id3, Msx1* and *Msx2* in sorted mammary myoepithelial cells from control and cKO mice at pregnancy day 14.5. n ≥ 3 biological replicates. Data were presented as means ± SD. $^{*}p < 0.05$, $^{**}p < 0.01$, $^{***}p < 0.001$.

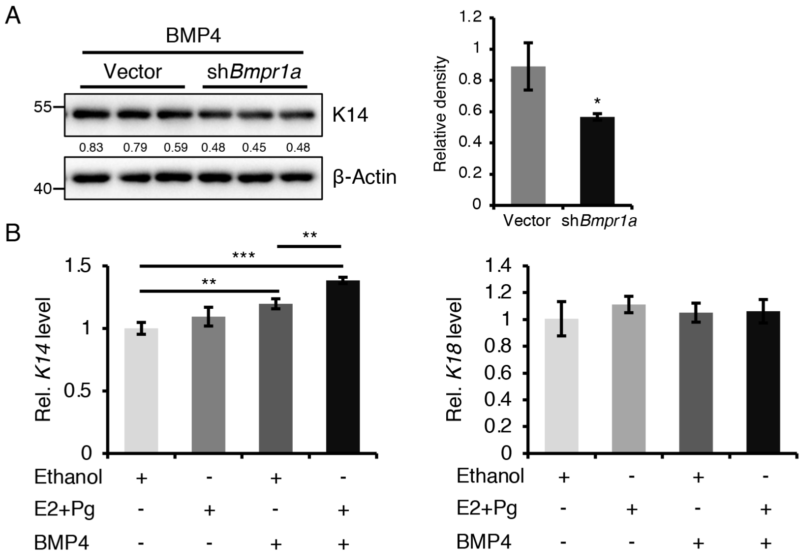

Figure S5. BMP signaling maintained myoepithelial cell lineage in HC11 cells

(A) HC11 cells transfected with control (Vector) and *Bmpr1a* knockdown (sh*Bmpr1a*) plasmids, then treated with BMP4 (50 ng/mL) for 24 h, western blotting analysis of K14 in the HC11 cells under indicated conditions. β-Actin was used as a loading control. Statistical analysis the expression of K14/β-Actin. n = 3 biological replicates. **(B)** HC11 cells were treated with E2 (10 nM) and Pg (100 nM) for 12 h, then stimulated with or without BMP4 (50 ng/mL) for 24 h, qRT-PCR analysis of *K14* and *K18* in the HC11 cells under indicated conditions. n = 3 biological replicates. Data were presented as means ± SD. $^{*}p < 0.05$, $^{**}p < 0.01$, $^{***}p < 0.001$.

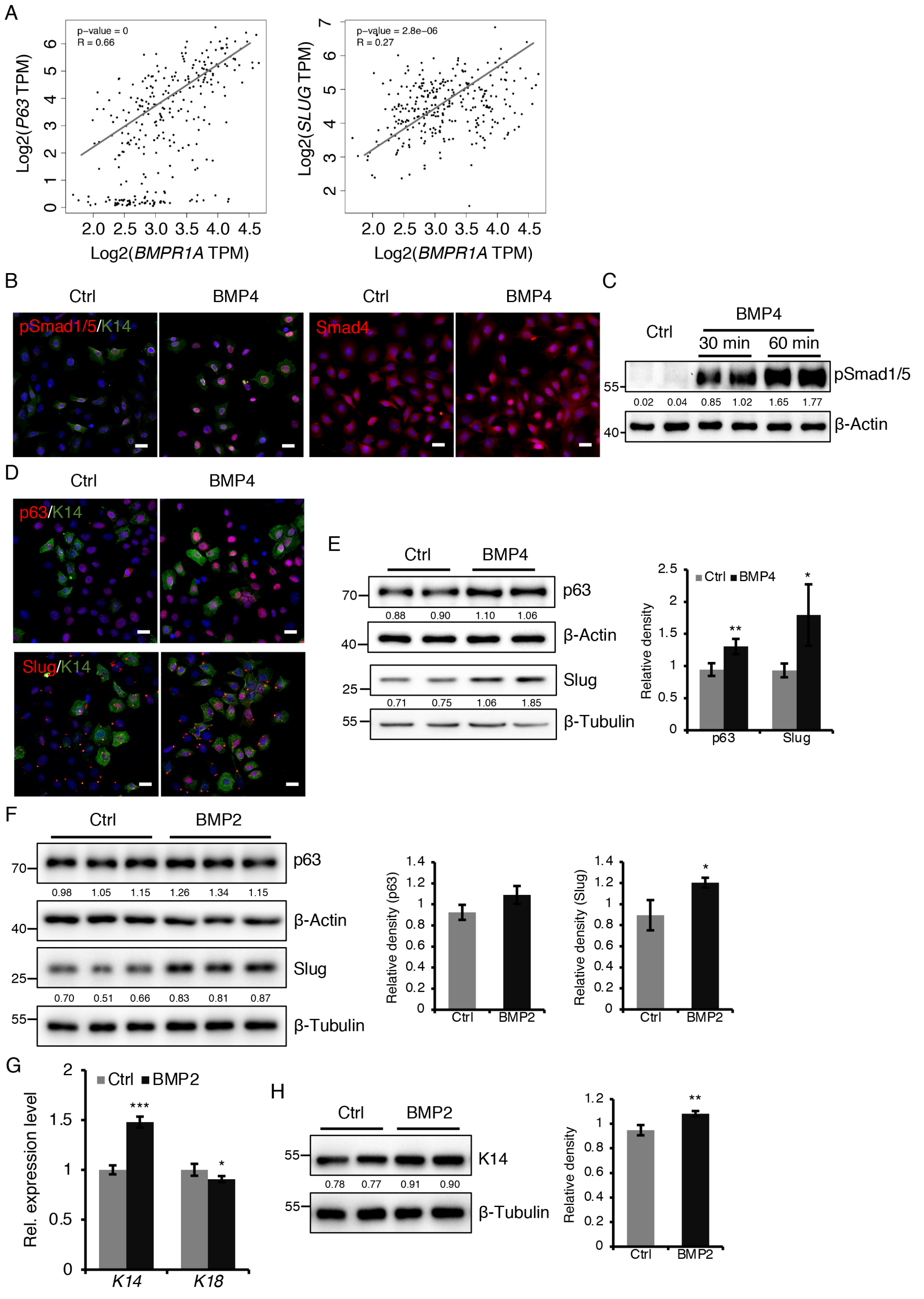

Figure S6. Activated BMP signaling upregulated p63 and Slug expression

(A) Scatter plot showing the correlation between *BMPR1A* and *P63* or *BMPR1A* and *SLUG* expression in mammary glands from TCGA and GTEx data. Pearson's coefficient test was performed to assess statistical significance. **(B)** Immunofluorescence for pSmad1/5 (red), Smad4 (red) and K14 (green) in HC11 cells

treated with BMP4 (50 ng/mL) for 1 h. n = 3 biological replicates. Scale bar, 25 μm. **(C)** Western blotting for pSmad1/5 in HC11 cells treated with BMP4 (50 ng/mL) at the indicated time points. β-Actin was used as a loading control. **(D)** Immunofluorescence for p63 (red) or Slug (red) and K14 (green) in HC11 cells treated with BMP4 (50 ng/mL) for 24 h. n = 3 biological replicates. Scale bar, 25 μm. **(E)** Western blotting for p63 and Slug in HC11 cells treated with BMP4 (50 ng/mL) for 24 h. β-Actin and β-Tubulin were used as loading controls. Statistical analysis the expression of p63/β-Actin and Slug/β-Tubulin. n = 4 biological replicates. **(F)** Western blotting for p63 and Slug in HC11 cells treated with BMP2 (50 ng/mL) for 24 h. β-Actin and β-Tubulin were used as loading controls. Statistical analysis the expression of p63/β-Actin and Slug/β-Tubulin. n = 3 biological replicates. **(G)** qRT-PCR analysis of *K14* and *K18* in HC11 cells treated with BMP2 (50 ng/mL) for 24 h. n = 4 biological replicates. **(H)** Western blotting for K14 in HC11 cells treated with BMP2 (50 ng/mL) for 24 h. β-Tubulin was used as a loading control. Statistical analysis the expression of K14/β-Tubulin. n = 4 biological replicates. Data were presented as means ± SD. $^{*}p < 0.05$, $^{**}p < 0.01$, $^{***}p < 0.001$.

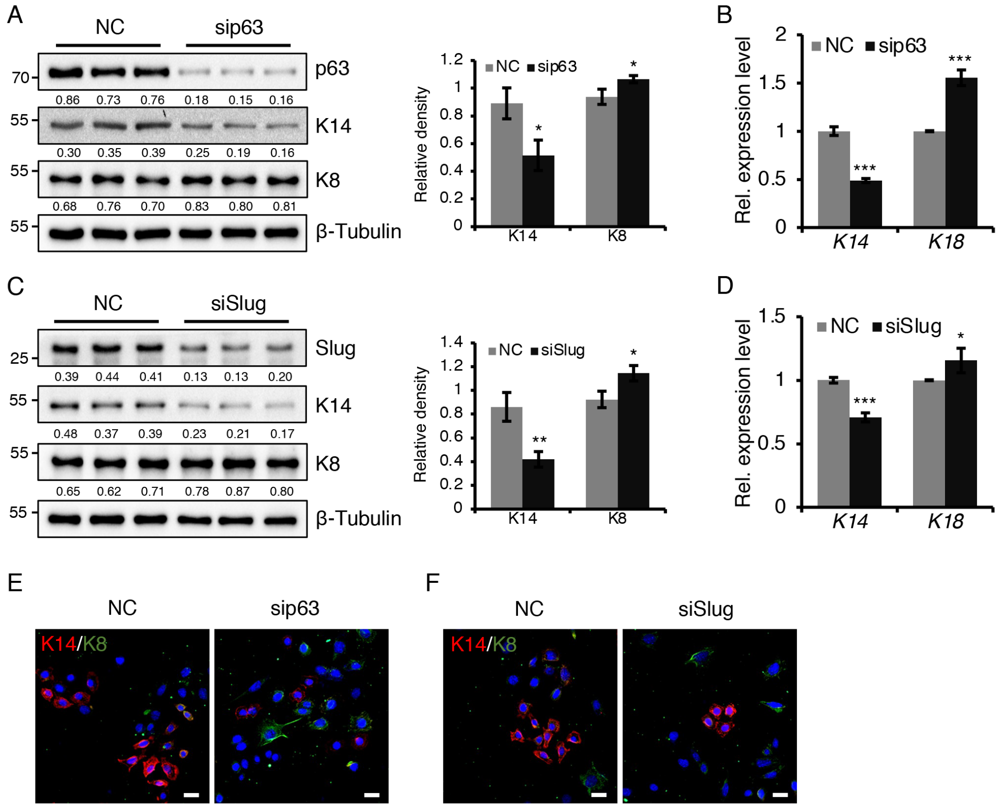

Figure S7. Knockdown of p63 or Slug resulted in a reduction in K14⁺ cells
(A) Western blotting for p63, K14 and K8 in HC11 cells treated with p63 siRNA (sip63) and scramble RNA (NC) at 48 h. β-Tubulin was used as a loading control. Statistical analysis the expression of K14/β-Tubulin and K8/β-Tubulin. n = 3 biological replicates. **(B)** qRT-PCR analysis of *K14* and *K18* in HC11 cells treated with p63 siRNA (sip63) and scramble RNA (NC) at 48 h. n = 3 biological replicates. **(C)** Western blotting for Slug, K14 and K8 in HC11 cells treated with Slug siRNA (siSlug) and scramble RNA

(NC) at 48 h. β-Tubulin was used as a loading control. Statistical analysis the expression of K14/β-Tubulin and K8/β-Tubulin. n = 3 biological replicates. **(D)** qRT-PCR analysis of *K14* and *K18* in HC11 cells treated with Slug siRNA (siSlug) and scramble RNA (NC) at 48 h. n = 3 biological replicates. **(E)** Immunofluorescence staining for K14 (red) and K8 (green) in HC11 cells treated with p63 siRNA (sip63) and scramble RNA (NC) at 48 h. n = 3 biological replicates. Scale bar, 25 μm. **(F)** Immunofluorescence staining for K14 (red) and K8 (green) in HC11 cells treated with Slug siRNA (siSlug) and scramble RNA (NC) at 48 h. n = 3 biological replicates. Scale bar, 25 μm. Data were presented as means ± SD. $^{*}p < 0.05$, $^{**}p < 0.01$, $^{***}p < 0.001$.

Supplementary Table 1. Primers for genotyping

**Gene**	**Sequences 5'-3'**
K14-rtTA Forward	CACGATACACCTGACTAGCTGGGTG
K14-rtTA Reverse	CATCACCCACAGGCTAGCGCCAACT
teto-Cre control Forward	CTAGGCCACAGAATTGAAAGATCT
teto-Cre control Reverse	GTAGGTGGAAATTCTAGCATCATCC
teto-Cre trans Forward	GCGGTCTGGCAGTAAAAACTATC
teto-Cre trans Reverse	GTGAAACAGCATTGCTGTCACTT
$Bmpr1a^{flox}$ Forward	GCAGCTGCTGCTGCAGCCTCC
$Bmpr1a^{flox}$ Reverse	TGGCTACAATTTGTCTCATGC
mTmG-1	CTCTGCTGCCTCCTGGCTTCT
mTmG-2	CGAGGCGGATCACAAGCAATA
mTmG-3	TCAATGGGCGGGGGTCGTT

Supplementary Table 2. siRNA/shRNA sequences

**siRNA/shRNA**	**Sequence sense 5'-3'**
NC	UUCUCCGAACGUGUCACGUTT
sip63	AUCGAUGCCGUGCGCUUUATT
siSlug	GGAGCAUACAGCCCUAUUATT
sh*Bmpr1a*	GCTGTCTGTATAGTTGCTATG

Supplementary Table 3. qRT-PCR primer sequences

Gene	Forward 5'-3'	Reverse 5'-3'
Bmpr1a	AACAGCGATGAATGTCTTCGAG	GTCTGGAGGCTGGATTATGGG
Bmpr1b	CCCTCGGCCCAAGATCCTA	CAACAGGCATTCCAGAGTCATC
Bmpr2	TTGGGATAGGTGAGAGTCGAAT	TGTTTCACAAGATTGATGTCCCC
Bmp2	GGGACCCGCTGTCTTCTAGT	TCAACTCAAATTCGCTGAGGAC
Bmp4	TTCCTGGTAACCGAATGCTGA	CCTGAATCTCGGCGACTTTTT
Wap	CGCTCAGAACCTAGAGGAACA	CGGGTCCTACCACAGGAAAC
Lalba	ATTTCGTTCCTTTGTTCCTGGT	TGCCTTGATAGCCATCTATGTCT
Csn2	GGCACAGGTTGTTCAGGCTT	AAGGAAGGGTGCTACTTGCTG
Elf5	ATGTTGGACTCCGTAACCCAT	GCAGGGTAGTAGTCTTCATTGCT
Tnfsf11	CAGCATCGCTCTGTTCCTGTA	CTGCGTTTTCATGGAGTCTCA
Src	GAACCCGAGAGGGACCTTC	GAGGCAGTAGGCACCTTTTGT
Socs-1	CTGCGGCTTCTATTGGGGAC	AAAAGGCAGTCGAAGGTCTCG
Socs-2	AGTTCGCATTCAGACTACCTACT	TGGTACTCAATCCGCAGGTTAG
Id1	CCTAGCTGTTCGCTGAAGGC	CTCCGACAGACCAAGTACCAC
Id2	ATGAAAGCCTTCAGTCCGGTG	AGCAGACTCATCGGGTCGT
Id3	CTGTCGGAACGTAGCCTGG	GTGGTTCATGTCGTCCAAGAG
Msx1	TGCTGCTATGACTTCTTTGCC	GCTTCCTGTGATCGGCCAT
Msx2	CAAGAAGCCGCCCAAGGAAT	TGCTCCGTCTTCGGAATTTTC
p63	CTGTACTGCCAGATTGCGAA	CTCATTGAACTCACGGCTCA
ΔNp63	GGAAAACAATGCCCAGACTC	GTGGAATACGTCCAGGTGGC

TAp63	TTACAGATCTGCCATGTCGC	CCCAGATATGCTGGAAGACC
Slug	TGGTCAAGAAACATTTCAACGCC	GGTGAGGATCTCTGGTTTTGGTA
K14	CCTCTGGCTCTCAGTCATCC	GAGACCACCTTGCCATCG
K18	AAGGTCTGGAAGCCCAGATT	CTTGGTGGTGACAACTGTGG
Cdh3	CTGGAGCCGAGCCAAGTTC	GGAGTGCATCGCATCCTTCC
Itgb3	CCACACGAGGCGTGAACTC	CTTCAGGTTACATCGGGGTGA
Adamts18	TGTCGTGCCAGTAGAAGTGG	AGCACCGTCCTTTCCAAGTA
Cdh2	TTACAGCTACCTGCCACTTTTC	CCAGCAGATTTCAAGGTGGAC
Tspan8	TCTGGGTATGTGGTACACTGAT	AGGGGTTCGTGCTAGAGTCTC
Lgr5	CGGGACCTTGAAGATTTCCT	GATTCGGATCAGCCAGCTAC
Procr	AATGCCTACAACCGGACTCG	ACCAGTGATGTGTAAGAGCGA
Cd44	CACCATTGCCTCAACTGTGC	TTGTGGGCTCCTGAGTCTGA
Spp1	TCCCTCGATGTCATCCCTGTTG	GGCACTCTCCTGGCTCTCTTTG
Fgf2	GCGACCCACACGTCAAACTA	TCCCTTGATAGACACAACTCCTC
Hgf	ATGTGGGGGACCAAACTTCTG	GGATGGCGACATGAAGCAG
Nrg1	ATGGAGATTTATCCCCCAGACA	GTTGAGGCACCCTCTGAGAC
Gapdh	TCCCACTCTTCCACCTTCGATGC	GGGTCTGGGATGGAAATTGTGAGG

Supplementary Table 4. Primer sequences for ChIP qPCR

Gene	Sequences 5'-3'
Slug-promoter Smad1 Forward	TGTCATCAGCCGGTGGACTTC
Slug-promoter Smad1 Reverse	ACCTGTGTGTTCCAGATTGTGC
Slug-promoter Smad4 Forward	TAGACCTGCTGTGGCAGC
Slug-promoter Smad4 Reverse	CCCTAGTGGCCAGAGAGTC
p63-promoter Smad4 Forward	AACAACTTCGTGTCCTGCCTG
p63-promoter Smad4 Reverse	CATAAGAAAGTGCACTCTGTCC
